# Australian Cool-Season Pulse Seed-Borne Virus Research: 1. Alfalfa and Cucumber Mosaic Viruses and Less Important Viruses

**DOI:** 10.3390/v16010144

**Published:** 2024-01-18

**Authors:** Roger A. C. Jones, Benjamin S. Congdon

**Affiliations:** 1 UWA Institute of Agriculture, University of Western Australia, Crawley, WA 6009, Australia; 2 Department of Primary Industries and Regional Development, South Perth, WA 6151, Australia; benjamin.congdon@dpird.wa.gov.au

**Keywords:** Australia, history, virus diseases, seed-borne viruses, epidemiology, management, losses, future challenges, research priorities

## Abstract

Here, we review the research undertaken since the 1950s in Australia’s grain cropping regions on seed-borne virus diseases of cool-season pulses caused by alfalfa mosaic virus (AMV) and cucumber mosaic virus (CMV). We present brief background information about the continent’s pulse industry, virus epidemiology, management principles and future threats to virus disease management. We then take a historical approach towards all past investigations with these two seed-borne pulse viruses in the principal cool-season pulse crops grown: chickpea, faba bean, field pea, lentil, narrow-leafed lupin and white lupin. With each pathosystem, the main focus is on its biology, epidemiology and management, placing particular emphasis on describing field and glasshouse experimentation that enabled the development of effective phytosanitary, cultural and host resistance control strategies. Past Australian cool-season pulse investigations with AMV and CMV in the less commonly grown species (vetches, narbon bean, fenugreek, yellow and pearl lupin, grass pea and other *Lathyrus* species) and those with the five less important seed-borne pulse viruses also found (broad bean stain virus, broad bean true mosaic virus, broad bean wilt virus, cowpea mild mottle virus and peanut mottle virus) are also summarized. The need for future research is emphasized, and recommendations are made regarding what is required.

## 1. Introduction

Worldwide, virus disease pandemics and major epidemics greatly diminish the vigor and growth of cultivated plants, decreasing their yields and produce quality [1,2,3,4,5,6,7]. Major trends, such as climate change, agricultural and trade globalisation, virus resistance breakdown and vector pesticide resistance, are increasing their spread and making their management more difficult globally [3,5,6,7,8,9,10,11,12,13,14,15,16,17]. This situation also applies to the Australian continent, which spans tropical, subtropical, Mediterranean and temperate climatic zones, and grows almost the entire range of cultivated species planted worldwide. Moreover, it still applies to Australia despite (i) the relatively recent appearance of agriculture involving introduced cultivated plants that followed the arrival of European colonists in 1788; (ii) the protective sea barrier from being an isolated island continent; and (iii) having one of the world’s most effective plant biosecurity systems [6,7,11,18,19,20,21,22,23,24,25]. A diverse array of legumes is present among the introduced, cultivated plant species that suffer from virus disease epidemics in Australia [18,26,27]. These include leguminous crops often called ‘pulses’ from which dry seeds with low fat content are harvested [28,29], the term ‘pulse’ coming from the Latin word “puls”, which literally means pottage (=thick soup) [30]. Pulse seeds have a high protein content and, therefore, provide a major dietary component for millions of people worldwide [31]. They are often grown with oilseeds and cereals in crop rotations. This is mainly because they fix nitrogen in the soil, thereby contributing to more profitable and sustainable farming [32,33].

In Australia, the principal pulses grown are narrow-leafed lupin (*Lupinus angustifolius),* white lupin (*Lupinus albus*), chickpea (*Cicer arietinum*), field pea (*Pisum sativum*), faba bean (*Vicia faba*), lentil (*Lens culinaris*) and mung bean (*Vigna radiata*), but common vetch (*Vicia sativa*), common and navy beans (*Phaseolus vulgaris*), cowpea **(***Vigna unguiculata*), adzuki bean (*Vigna angularis*) and pigeon pea (*Cajanus cajan*) are also grown [33,34,35,36]). In addition, pearl lupin (*Lupinus mutabilis*), yellow lupin (*Lupinus luteus*), sandplain lupin (*Lupinus cosentinii*), grass pea (*Lathyrus sativus*), dwarf chickling (*Lathyrus cicera*), narbon bean (*Vicia narbonensis*), bitter vetch (*Vicia ervilia*) and fenugreek (*Trigonella foenum-graecum*) are examples of other pulses suited to Australian conditions. These are sometimes grown for grain or forage currently and are available for wider use in the future [34,36,37,38,39,40,41,42]. However, pulses exclude introduced legume crops classified as oilseeds, e.g., peanut (*Arachis hypogaea*) and soybean (*Glycine max*). This is because they produce seeds with a high fat content so are grown mostly for oil extraction. Pulses also exclude legumes grown as vegetables, such as green peas and green beans, as their pods are harvested and eaten when still immature [28,29]). Notably, the cultivated legumes that suffer from virus disease epidemics also include introduced plants widely sown in the managed pastures that support Australia’s extensive meat, dairy and wool industries. Furthermore, compared with pulses, pasture legume species are commonly present for longer periods during the annual growing season and, in some cases, are perennials. They, therefore, constitute an important virus reservoir for the spread of legume viruses into pulse crops [18,27,43,44,45,46,47]).

The books ‘Viruses of Legumes 1983′ [26] and ‘Viruses of Tropical Plants’ [48], which both have Australian authors or co-authors, provided brief accounts of what was known about virus diseases of grain and pasture legumes in Australia up to 1982 and 1989, respectively. The book ‘Viruses of Plants in Australia’ [18] provided more detailed information about legume viruses in the Australian continent up to 1987. A global review of lupin virus disease research written by Australian authors included information about virus disease issues in this pulse in Australia up to 1988 [49]. In addition, a global review of research on the control of virus diseases in cool-season pulses up to the year 2013 included information from Australia and had Australian co-authors [50]. Cool-season pulses (e.g., chickpea, faba bean, field pea, lentil and lupin) differ from those adapted to warmer conditions (e.g., common bean, cowpea, mung bean, pigeon pea) in that they are also well adapted to cooler conditions. A more recent global review focused specifically on host resistance to, and breeding for resistance against, lupin virus diseases also included information from Australia [51]. The book ‘Diseases of Vegetable Crops in Australia’ published in 2010, included an account of virus diseases of green peas and green beans [52]. A more detailed review focused on the development of integrated disease management strategies against two lupin virus diseases in Australia up to the year 2000 [44]. Also, a general review of Australian research on pathogens of cool-season pulses briefly summarised virus disease findings up to the year 2010 [27]. More recently, a comprehensive review of Australian research on oilseed and cereal virus diseases up to the year 2020 included information on viruses infecting the oilseed legumes, soybean and peanut [24]. Also, a recent review entitled ‘enhancing biosecurity against virus disease threats to Australian grain crops: current situation and future prospects’ provided details of current pulse crop biosecurity threats [25]. Furthermore, several reviews described past Australian pasture legume research: (i) general Australian pasture pathogen disease reviews included summaries of virus disease studies up to when they were published [53,54,55,56,57]); (ii) a more detailed review of research on subterranean clover (*Trifolium subterraneum*) virus diseases up until the year 1986 [58]; (iii) three reviews focused on findings with virus diseases of annual and perennial pasture legumes up until the year 2012 [43,45,46]; and (iv) a more recent review included Australian research about alterations to the species balance arising from virus infection of managed pastures mostly sown with pasture legume and/or grass species mixtures [47]. However, a comprehensive review of Australian seed-borne pulse virus disease research since the 1950s, like that recently published for virus diseases of oilseeds and cereals [24], is lacking despite the considerable volume of research performed on this subject over the last seven decades.

Cool-season pulses and other cultivated legumes often become infected by seed-borne viruses, which cause economically important diseases. Indeed, whether a virus is or is not seed-borne has major implications for its introduction to new localities, and for its ecology, epidemiology, and management [15,16,44,50,59,60,61,62,63,64]. Seed transmission enables a virus to survive in dormant seeds in periods when its annual plant hosts are unable to grow, e.g., during cold winter or hot, dry summer conditions. Following their germination, the seed-infected plants provide a primary inoculum source for virus spread by vector or contact transmission. Managing epidemics of seed-borne viruses in pulse crops requires sowing healthy seeds or seeds with low levels of seed-borne infection [15,16,50,59,60,61,62,63,64]. Movement of virus-infected seeds by human activity, or even inside the intestines of animals, enables virus introductions both nearby or further away, including between other continents and Australia [11,19,20,25]. Preventing the introduction of seed-borne viruses of pulses to new countries or continents, such as Australia, requires rigorous biosecurity measures [22,23,25].

Here, we provide the first volume of a three-part series of comprehensive review articles that take a historical approach towards describing seven decades of research across Australia’s different agroecological zones on seed-borne virus diseases of cool-season pulse crops. Four seed-borne viruses cause diseases that seriously threaten cool-season pulse crop production in Australia. These are alfalfa mosaic virus (AMV; genus *Alfamovirus*, family *Bromoviridae*), bean yellow mosaic virus (BYMV; genus *Potyvirus*, family *Potyviridae*), cucumber mosaic virus (CMV; genus *Cucumovirus*, family *Bromoviridae*) and pea seed-borne mosaic virus (PSbMV; genus *Potyvirus*, family *Potyviridae*) [18,44,50,60,62]. In addition, five other seed-borne viruses of cool-season pulses have been found but currently are of minor economic importance. These are broad bean stain virus (BBSV; genus, *Comovirus*, family *Secoviridae*), broad bean true mosaic virus (BBTMV; genus, *Comovirus*, family *Secoviridae*), broad bean wilt virus (BBWV; genus *Fabarvirus*, family *Secoviridae*), cowpea mild mottle virus (CMMV; genus *Carlavirus*, family *Betaflexiviridae*) and peanut mottle virus (PMoV; genus *Potyvirus*, family *Potyviridae*) [18,65,66]). We commence this volume by providing brief general background information about the Australian pulse industry, the seed-borne viruses so far found infecting the continent’s cool-season pulse crops, virus epidemiology, management principles and future threats to pulse virus disease management. Thereafter, we provide a detailed historical account of the knowledge accumulated concerning the biology, epidemiology and management of the cool-season pulse/virus pathosystems that involve infection with AMV and CMV. Next, brief coverage is provided of past Australian studies with the five minor viruses of cool-season pulses. This volume also makes recommendations regarding what future research is required in Australia.

## 2. Background Information

### 2.1. Australian Pulse Industry

**Figure 1:**
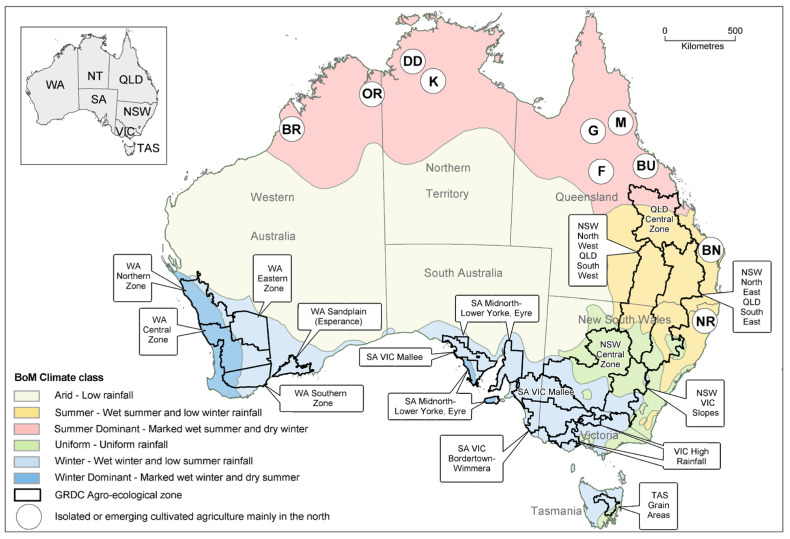
The acronyms of Australian states and territories used here are WA (Western Australia), SA (South Australia), VIC (Victoria), TAS (Tasmania), NSW (New South Wales), QLD (Queensland) and NT (Northern Territory). Australian Bureau of Meteorology (BOM) climate groupings are used to distinguish the main regions where grain crops grow, but letters distinguish the smaller irrigated grain production regions in WA, BR—Broome and OR—Ord River Irrigation Area; the NT, DD—Douglas/Daly and K—Katherine; QLD, BU—Burdekin, BN—Bundaberg, G—Gilbert, F—Flinders and M—Mareeba (including Atherton and Ravenshoe); NSW, NR—Northern Rivers. Black lines delineate boundaries of distinct agro-ecological zones (a modified version of those employed by the Australian Grains Research and Development Corporation (GRDC) [79]. Image reproduced from Jones et al. [24]. Image credit @Department of Primary Industries and Regional Development/P. Goulding.

About 22 million hectares of land are sown with crops annually in the Australian grainbelt, where cropping is highly mechanised with relatively low labour inputs and is rain-fed rather than relying on irrigation [67,68,69]. This grainbelt is located in south to central Queensland (QLD), New South Wales (NSW), Victoria (VIC), South Australia (SA) and southwest Western Australia (WA), and also includes the island state of Tasmania (TAS), which has a small grain-producing region (Figure 1). In eastern VIC, TAS and southern NSW, its climate is temperate, and in western VIC, SA and southwestern WA, it is Mediterranean, but in central and northern NSW and southeastern QLD, it is subtropical [68,70]. The amount of annual rainfall varies in the grainbelt’s cropping zones: 225–175 mm/year (low), 450–225 mm/year (medium) and 700–450 mm/year (high). The lowest seed yields occur in low rainfall zones where drought conditions occasionally cause complete crop failure [71,72,73]. A large proportion of Australian pulse production is exported for human consumption internationally, although lupin is mostly sold for animal feed. The average annual production of pulses is ~2.2 million metric tonnes, which is produced over >1.8 million hectares. However, there is potential to increase the area of pulses sown annually to 4.2 million hectares [74]. Because of their nitrogen fixation ability, such an area increase would also help by reducing nitrogen fertiliser requirements for non-legume crops grown afterward in the same fields. Moreover, in crop rotations involving cereals or oilseeds, pulse crops also act as ‘break’ crops because they provide a fungal disease, pest and weed break between successive growing seasons [32,33]. The principal pulse crops grown in the northern grainbelt (central and southern QLD and north and central-north NSW) are mungbean in summer, and chickpea, lentil, field pea and lupin in winter. Those grown in winter in the southern grainbelt (south and central south NSW, VIC, SA, TAS and WA) are field pea, faba bean, lupin, chickpea and lentil. Regional differences occur, however, e.g., in WA, lupin is a much bigger crop than in other regions, TAS grows no chickpea, central QLD does not grow lupin, field pea or faba bean, and in NSW, only its central north grows lupin [24,68,75]. Minor pulse crops sometimes grown in the Australian grainbelt include common vetch in all its climatic zones and common or navy beans, cowpea, adzuki bean and pigeon pea in the northern grainbelt. In addition, some irrigated pulse production occurs in tropical northern Australia: (i) in QLD in Burdekin, Flinders, Gilbert and Mareeba (including Ravenshoe and Atherton); (ii) in the Northern Territory (NT) in the Katherine and Daly/Douglas regions; and (iii) in the Ord River Irrigation Area in WA’s Kimberley region. The main pulses grown in these localities are common bean, chickpea, cowpea and mungbean, but pigeon pea is sometimes planted [76,77,78]. The tonnage of each of these individual crops harvested in each region varies widely from year-to-year depending on climatic factors, especially rainfall and market prices.

### 2.2. Epidemiology and Management Principles

A recent review of virus diseases of oilseed and cereal crops growing under the Australian continent’s diverse environmental conditions described what was known then about the underlying influences driving their epidemics and how to optimise their management [24]. However, since many factors affect the interactions that occur between virus, vector and plant host(s), understanding the epidemiology of each individual pathosystem and the factors driving its epidemics is critical if its management is to be effective. In brief, the extent of virus spread that occurs and the disease symptoms that result depend greatly upon whether the primary virus infection source is external or internal, the effect of differing weather conditions upon vector and virus biology, the type of vector and number of vector species involved, and whether vector transmission is non-persistent or persistent. Other critical factors include whether the temporal spread pattern is monocyclic or polycyclic and whether the spatial spread pattern is clumped or random [5,10,80]. Furthermore, when individual control measures are deployed on their own, these rarely provide effective control. Despite this, integrated disease management (IDM) approaches developed against virus diseases of grain legumes in Australia are capable of providing solutions that are not only sustainable but also socially, environmentally and economically viable. Devising them requires knowledge of factors that drove past Australian virus epidemics and, for each individual control measure, how it operates and how effective it is. With each IDM approach, virus-resistant cultivars (when available) are deployed in combination with cultural practices and phytosanitary measures that diminish virus spread or reduce the original virus source and pesticides that operate against vectors in an environmentally responsible way [5,9,10,44,50,62]. Moreover, exciting prospects for pre-emptive virus disease control are likely to arise from deploying new technologies and methodologies. For example, by combining precision agriculture with emerging technologies involving optical sensors and artificial intelligence to assist with identifying virus-diseased plants, establishing vector and virus disease occurrence, predicting potential virus disease-induced yield losses and selectively targeting localized virus infection and vector foci within crops with chemical control measures (herbicides or pesticides) instead of needing to treat entire crops [5,81,82,83,84,85]. Similarly, deploying speed breeding [86] and genetic modification using methods such as RNAi and CRISPR is likely to improve virus resistance options in commercial cultivars [87,88,89,90]. However, optimising the effectiveness of new technologies, methodologies and approaches still requires their integration with knowledge of the epidemiology of each individual pathosystem across the diverse range of agro-environments in which they are to be deployed [5,81].

### 2.3. Future Threats to Effective Virus Management

Future threats to controlling damaging Australian seed-borne pulse virus disease epidemics resemble those discussed for oilseed and cereal virus diseases in 2021 [24]). In brief, these threats were (i) the escalating difficulties in managing them due to increasing occurrence of extreme weather events and climate instability in general; (ii) the likely impact of alterations in agricultural practices and technologies, and other alterations in prevailing circumstances related to climate change (e.g., farmers shifting to carbon trading, increasing livestock production and other regenerative agriculture practices), or not necessarily related to climate change (e.g., adopting cultural practice advances, economising on inputs and greater use of drones, remote sensing and precision agriculture), causing shifts in virus and vector prevalence and the ability to control virus spread effectively; (iii) the increase in insect vector insecticide resistance and the likelihood that certain insecticides that still work effectively against vectors will be banned in the future; (iv) the appearance or inadvertent selection of virus strains that overcome host resistances currently in use in virus-resistant cultivar breeding; (v) the serious biosecurity risk arising from threatening viruses and virus vector species likely to invade from other continents; and (vi) the absence of adequate industry awareness concerning the economic threat posed by virus diseases. Among these factors, the threat from insecticide resistance in virus vectors and the future withdrawal of effective insecticides do not apply to their control in commercial pulse crops. This is because not only do the rapid virus acquisition times that typify non-persistently transmitted viruses like AMV and CMV render single or few insecticide applications ineffective at suppressing their spread [16,50], but also repeated regular (i.e., 2 weekly) application to kill insect vectors is not only impractical but also too expensive so is not a viable option. Nevertheless, such frequent regular insecticide applications to kill aphid vectors can help protect breeding programs and high-value seed crops such as newly released cultivars from CMV and AMV spread and virus contamination of their harvested seed. In 2023, the major biosecurity threat should further seed-borne viruses pulse viruses and their vectors arrive from other counties was described in detail, including recommendations for future research needs to counter this threat [25].

Three additional threats that need to be emphasised are the lack of (a) sufficient traditional research involving field experiments determining yield losses under conditions resembling those occurring in the field, providing information on the temporal and spatial dynamics of virus spread and establishing the effectiveness of different types of control measures; (b) regular annual surveillance to ascertain the incidence of virus infection in seed stocks and crops, and the occurrence of vector populations, in different cropping regions; and (c) research institution succession planning to ensure sufficient virology research capacity and expertise is available to address future virus disease outbreaks.

### 2.4. Recent General Recommendations for Future Research

Recommendations for future research relevant to all virus diseases of pulses were listed in 2018 in a grains industry status report on Australian pulse disease epidemiology and management [91]:

- As average temperatures increase and growing season rainfall rises or declines becoming more erratic, the climate of Australia’s grainbelt is changing fast. The weather is becoming increasingly unstable, causing a greater level of uncertainty about epidemics of virus diseases that increasingly compromises taking decisions over when control measures are required and, if so, which measures to deploy. Further research is therefore required to get ready for future pulse virus disease epidemics induced by climate change. For the viral pathogens identified as most likely to be affected, this will necessitate research addressing (a) the influence of climate change parameters (extreme weather events, insufficient rainfall, increased temperatures and wind speeds) upon their epidemiology and (b) the escalating difficulties in managing them effectively.
- Further research is required to establish how altering agricultural practices and technologies, and other alterations in prevailing circumstances unrelated to climate change, might influence virus disease epidemics occurring in pulse crops and, in turn, virus disease control strategies in the continent’s grainbelts. This includes (a) updating information on formerly better-studied pulse virus diseases and (b) addressing new virus disease problems of pulses likely to arise due to shifts in viral pathogen prevalence arising from farming system changes, cultivars or new virus strain and vector introductions.
- Ongoing annual surveillance for viruses infecting pulses and their vectors still needs to occur across the Australian grainbelt. This provides an understanding of their economic importance to prioritise research funding, an early warning of their occurrence across different growing seasons and rainfall zones, and the relative performances and robustness of any disease resistances where known.
- The use of historical data and new research to optimise modelling and decision support systems (DSSs) for virus diseases is crucial and needs to be expanded. With the better-researched pulse virus diseases, the principal focus should be on ensuring that all forecasting models and DSSs already available are updated to incorporate shifts in climatic, agronomic and pathosystem drivers since their initial development and delivery of DSSs derived from such models to the national pulse industry. For viral pathosystems without such models, further epidemiology and management research that complements existing findings is needed, leading to the development of improved, locally relevant forecasting models and DSSs and their delivery nationally.
- Molecular approaches to viral resistance in cool-season pulses warrant more support, especially those involving RNAi and CRISPR/Cas approaches.
- There is a clear need to ensure that the research capacity to respond to unforeseen circumstances involving pulse virus pathogens is maintained. This is necessary to ensure the pulse industry avoids being caught out by the absence of necessary expertise to address future unforeseen epidemics of virus diseases seriously affecting pulse crops.

Additional important general pulse virus disease recommendations not listed in the grains industry status 2018 report [61] are as follows:

- It is important to understand what biological and genotypic diversity is present within the economically important viruses of the different pulses that occur across Australia. This requires a combination of biological data derived from field and glasshouse experiments with whole genome sequencing of representative virus isolates from pulses followed by their phylogenetic analysis. Although much relevant biological data from Australia is available for AMV and CMV, information about the phylogenetics of a representative spectrum of complete isolate sequences of these two viruses from different pulses around Australia is lacking.
- It is important to keep up to date with new technologies that improve the efficiency of large-scale routine virus testing and diagnosis in pulse leaf and seed samples and the effectiveness of remote sensing technologies for virus disease and virus vector surveillance in the field using thermal, hyperspectral, multispectral and other remote sensing procedures.

### 2.5. Viruses Infecting Australian Pulse Crops

In Australia, a range of virus diseases decrease the value of pulse crops by (i) reducing both seed quality and yield and (ii) suppressing their nitrogen fixation and, as a result, its contribution to soil fertility for future non-legume crops. At the pulse industry level, the economic significance of a virus disease is determined by how common it is, the scale of the losses that occur and the value of the affected crop species. When virus symptoms are mild, but the virus disease occurs commonly and at high infection incidences, the losses may still be important economically. By contrast, if a virus disease is uncommon but associated with severe symptoms, it may be of little importance. As with cereal and oilseed crops infected by virus diseases [24], in individual pulse crops, the main factors determining the extent of virus-induced losses consist of: (i) infection incidence; (ii) cultivar susceptibility and sensitivity to infection; (iii) virulence of the strain(s) present; (iv) vector population arrival time and magnitude; (v) presence of other abiotic and biotic stressors; (vi) environmental conditions; and (viii) interactions between these factors. The cool-season pulse crops infected by seed-borne viruses in Australia and which grainbelt regions they are grown in, the worst recorded percentage yield losses, and the associated seed quality defects involved are shown in Table 1, which also lists the principal virus symptoms, the insect or mite vectors involved and in which growing regions they occur.

The greatest seed yield losses recorded from infection with AMV, BYMV, CMV or PSbMV in pulses are 99% (BYMV), 98% (AMV), 90% (CMV) and 96% (PSbMV) (Table 1). The recent review of virus diseases of oilseed and cereal crops in Australia described the ways information on seed yield and quality losses are obtained [24]. The same general principles also apply to seed-borne viruses of pulses. In brief, worst-case scenario yield loss information is often obtained by comparing the yield and quality of seeds from healthy and virus-infected plants growing in the glasshouse, outside in pots or as spaced plants or within single-row plots growing in the field. However, replicated field experiments with large plots where the virus spread between plants occur naturally provide the most representative data. This is because new infections occur at differing plant growth stages, and healthy plants growing next to stunted infected plants exhibit compensatory growth (i.e., occupy space that would otherwise be filled by neighboring plants if they were uninfected). The losses recorded are greatest when new infections occur at vulnerable early plant growth stages, the virus source is evenly distributed across the whole experiment, and wide non-host crop barriers minimize virus spread into healthy control plots. Meaningful seed yield and quality loss data from past large-scale field experiments with pulse crops are available from Australia. With the host–virus combinations in Table 1, such field experiments include ones where the infecting seed-borne virus was CMV in lupin and chickpea, BYMV in lupin and PSbMV in field pea [92,93,94,95,96,97,98,99,100,101].

## 3. Cucumber Mosaic Virus

**Figure 2:**
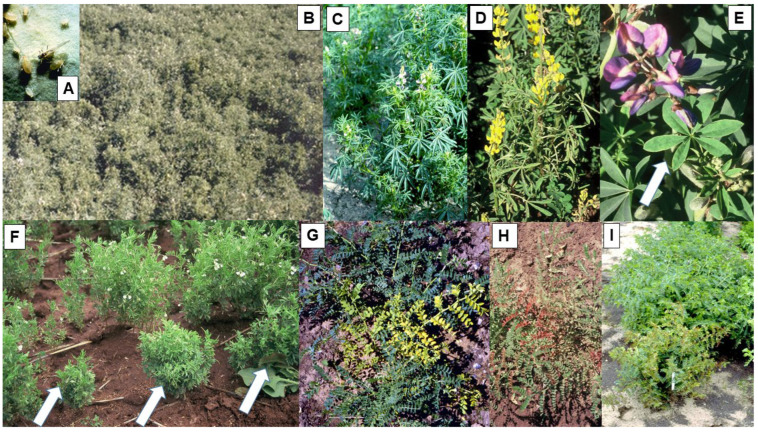
The critically important aphid vector *Myzus persicae* and examples representing the diversity of current-season symptoms that cucumber mosaic virus (CMV) infection elicits in the foliage of different cool-season pulse plant species. (**A**), Winged and unwinged individuals of *M. persicae*. (**B**), Typical appearance of a narrow-leafed lupin crop with 100% CMV infection, its uneven crop canopy resulting from plants with different extents of dwarfing due to infection at different plant growth stages (Corrigin, 1993). (**C**), Narrow-leafed lupin plant with typical foliage symptoms of leaflet size reduction, downcurling and chlorosis in new growth and plant dwarfing but with normal-looking lower leaves (front), healthy plants (back) (Wongan Hills, 1995). (**D**), Yellow lupin plant with typical foliage symptoms of leaflet size reduction, faint mottle, chlorosis and downcurling in new growth and overall plant dwarfing (front), healthy plants (back) (South Perth, 1993). (**E**), Upper portion of the pearl lupin plant showing leaf with mosaic indicated by a white arrow (South Perth, 1993). (**F**), Lentil plants showing leaflet symptoms of pallor and size reduction, and overall plant dwarfing indicated by a white arrow (front), healthy plants (behind) (Cunderdin, 1997). (**G**), Plant of Kabuli chickpea with typical foliage symptoms of leaflet chlorosis (centre), healthy plants (above and below) (South Perth, 1997). (**H**), Plants of desi chickpea with typical foliage symptoms of leaflet reddening (South Perth, 1997). (**I**), Plant of desi chickpea showing leaflet reddening and chlorosis (front), healthy plants (back) (South Perth, 1996).

CMV was first described in 1916 and was one of the earliest plant viruses studied [102]. When it first arrived in Australia is unknown, but the first Australian research publications that mention it were in 1954 [103,104]. It has a very wide host range consisting of >1200 species within >100 dicotyledonous and monocotyledonous families of flowering plants and is non-persistently transmitted by >80 aphid species among which *Myzus persicae* (the green peach aphid; Figure 2A) and *Aphis gossypii* (the cotton aphid) are critically important [102]. CMV occurs in all Australian states and territories. Pulses and pasture legume weeds are among the many economically significant cultivated plants that CMV infects in Australia. The pulse hosts include narrow-leafed lupin, yellow lupin, pearl lupin, lentil, chickpea, field pea, faba bean, narbon bean, grass pea, dwarf chickling and fenugreek (Table 1; [18,105,106,107,108]. In infected pulse species, the foliage symptoms it elicits vary widely but typically involve leaf mosaic, chlorosis and deformation, and plant dwarfing (Table 1; Figure 2B–I). An extensive list of early records (mid-1950s–mid-1980s) of CMV infecting crop, pasture and weed host species from QLD, NSW, VIC, SA, TAS and WA was provided by Buchen-Osmond et al. [18].

**Table 1:** Seed-borne viruses infecting cool-season pulse crops in Australia.

Virus	*Virus Genus*	Pulse Crops Affected (Includes Non-Cool-Season Pulses)	Main Foliage Symptoms	Vector	Seed-Borne	Maximum Yield Loss	Seed Quality Defect	Region Found In ^a^
Most important viruses								
Alfalfa mosaic virus (AMV)	*Alfamovirus*	Chickpea, field pea, faba bean, lentil, narrow-leafed lupin, yellow lupin, narbon bean, mung bean, common vetch, Adzuki bean, fenugreek, narbon bean, grass pea, dwarf chickling, purple vetch, *Lathyrus ochrus*	Mosaic, leaf deformation, dwarfing	Aphid	Yes	98%	Reduced size	STE, MED, TE
Bean yellow mosaic virus (BYMV)	*Potyvirus*	Chickpea, common bean, faba bean, field pea, lentil, narrow-leafed lupin, white lupin, yellow lupin, sandplain lupin, pearl lupin, common vetch, bitter vetch, narbon bean, grass pea, dwarf chickling, fenugreek	Mosaic, leaf deformation, dwarfing	Aphids	Yes	99%	Reduced size (necrosis and malformation in faba bean)	STE, MED, TE
Cucumber mosaic virus (CMV)	*Cucumovirus*	Chickpea, faba bean, field pea, lentil, narrow-leafed lupin, yellow lupin, pearl lupin, narbon bean, fenugreek, bitter vetch	Mosaic, chlorosis, leaf deformation, plant dwarfing	Aphids	Yes	90%	Reduced size	STE, MED, TE
Pea seed-borne mosaic virus (PSbMV)	*Potyvirus*	Chickpea, faba bean, field pea, lentil, common vetch, fenugreek, narbon bean, dwarf chickling, grass pea, bitter vetch, purple vetch, *L. clymenum*, *L. ochrus*	Mosaic, mild dwarfing	Aphids	Yes	96%	Necrotic rings, malformation, cracking, reduced size	STE, MED, TE
**Less important viruses**								
Broad bean stain virus (BBSV)	*Comovirus*	Faba bean, lentil, field pea, common vetch	Mottle, leaf deformation, necrosis	Beetle	Yes	61%	Reduced seed size, necrosis, malformation	TE
Broad bean true mosaic virus (BBTV)	*Comovirus*	Faba bean, lentil, field pea, common vetch	Mottle, leaf deformation, necrosis	Beetle	Yes	30%	Reduced seed size, necrosis, malformation	TE
Broadbean wilt virus 2 (BBWV-2)	*Fabavirus*	Faba bean, chickpea, field pea, narrow-leafed lupin, white lupin, cowpea, common bean	Vein clearing, mottle, leaf deformation, apical necrosis, ringspot, wilting	Aphid	Yes	26%	Reduced seed size, necrosis, malformation	STE, TE
Cowpea mild mottle virus (CpMMV)	*Calarvirus*	Mungbean, common bean, lima bean, field pea, cowpea	Mosaic, leaf distortion, necrosis, dwarfing, pod distortion	Whitefly	Yes	100%	Reduced seed size	STE
Peanut mottle virus (PMoV)	*Potyvirus*	Common bean, lima bean, Adzuki bean, cowpea, field pea, narrow-leafed lupin, white lupin	Mottle, necrosis	Aphid	Yes	70%	Reduced size, malformation, discoloration	STE

Information sources: Buchen-Osmond et al. (1988) [18], AAB Descriptions of Plant Viruses [109], CABI Data Sheets [110], VIDE Data Base [48], Searches using Google and Google Scholar. ^a^ Australian grain growing regions: tropical north (TN), subtropical east (STE), Mediterranean (MED) and temperate (TE).

### 3.1. Lupins

#### 3.1.1. Breeding Program and Commercial Seed Stock Contamination

**Figure 3:**
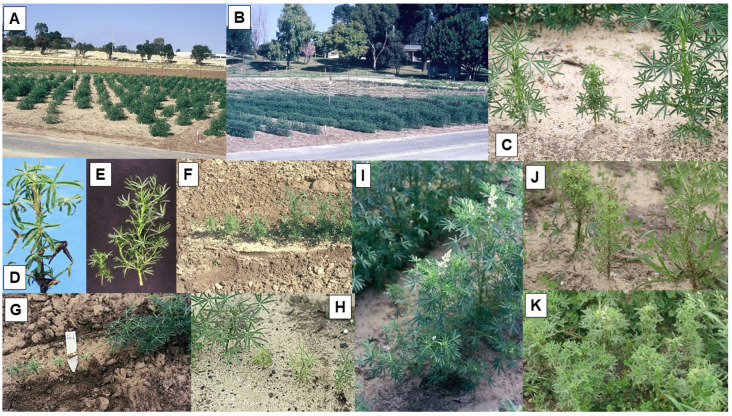
Cucumber mosaic virus infection of single-row narrow-leafed lupin breeding plots growing at South Perth in 1986. (**A**), Block of short single-row F1-generation plots. (**B**), Single-row plots showing uneven growth due to CMV spread from seed-infected to current-season-infected plants, most evident in the first 4 rows (bottom left). (**C**), Part of F1-generation progeny single-row lupin plot showing young seed-infected plant (centre) with healthy plants on either side; seed-borne infection caused leaflet size reduction, chlorosis and downcurling and plant dwarfing. (**D**), close up of F1 lupin seedling plant with seed-borne infection. (**E**), Size comparison between seed-infected F1 seedling (left) and much taller healthy F1 seedling of the same age (right), seed-infected seedling plant severely dwarfed. (**F**), F1-generation single-row lupin plot showing early CMV spread from small seed-infected seedling (furthest left) to the two nearest seedlings but not to normal-looking seedlings further to the right. (**G**), Single-row plot of wild lupin germplasm accession showing early CMV spread from tiny seed-infected seedling (next to white pot label) to chlorotic dwarfed seedlings on its left but not to normal-looking healthy seedlings on its right. (**H**), Single-row plot of wild lupin germplasm accession showing early CMV spread from tiny seed-infected seedling (centre) to dwarfed seedlings on its right but not to normal-looking healthy seedlings on its left. (**I**), End plant of single-row F1-generation lupin plot with interplot CMV spread showing typical late current-season foliage symptoms consisting of leaflet chlorosis, reduced size and downcurling of young leaves combined with normal-looking lower leaves. (**J**), Volunteer plants: seed-infected plant at centre with all leaves showing chlorotic symptoms, current-season-infected plant behind has foliage symptoms in its apical leaves but normal-looking lower leaves, unaffected volunteer plant on the right side. (**K**), Volunteer lupin plants with typical current-season CMV symptoms (leaflet chlorosis, size reduction and downcurling, and plant dwarfing) in apical growth.

After the first Australian narrow-leafed lupin cultivar with minimal seed alkaloid content was released in 1967 [111], lupin cultivation increased rapidly, with 900,000 ha sown annually by 1987 in the southwest WA grainbelt [112], and smaller areas sown in SA, NSW and VIC. In Australia, CMV was first detected infecting narrow-leafed lupin in 1978 in NSW and 1979 in WA [113]. By the early 1980s, it was causing damaging virus epidemics in narrow-leafed lupin crops in WA, SA and NSW. The foliage symptoms it elicited in the field were mosaic, chlorosis, leaflet bunching and plant dwarfing and seed production was diminished (Figure 2B,C) [96,99,112,114,115,116,117]. In 1986, the Australian national narrow-leafed lupin program breeding plots in WA were found to be severely CMV affected (Figure 3A–K), and the newly released cv. Wandoo was withdrawn because of severe damage from CMV infection. Evidence that CMV was being spread by sowing infected narrow-leafed lupin seed followed by aphid transmission from seed-infected plants to healthy plants was soon demonstrated, and widespread contamination of Australian breeding lines and commercial seed stocks was revealed [112,116]. Earlier studies in the USA and South Africa, and in SA in Australia, had also found CMV to be seed-borne in narrow-leafed lupin [115,118,119]. In WA, levels of seed transmission to seedlings detected varied widely with breeders’ selection, cultivar and seed stock. When seedlings from samples from 28 seed stocks of three new cultivars were tested, the virus was detected in 10/10 (cv. Wandoo), 6/8 (cv. Gungurru) and 5/10 (cv. Danja) seed stocks. Also, when seed stocks of 18 established cultivars were tested, the corresponding figures were 6/12 (cv. Illyarrie), 3/4 (cv. Yandee) and 2/2 (cv. Chittick). The CMV seed-transmission rates to seedlings found in samples from different infected seed stocks were up to 34% (Wandoo), 18% (Illyarrie), 15% (Danja), 8% (Chittick), 5% (Yandee) and 2% (Gungurru). When cv. Wandoo seed with 34% seed transmission was sieved to separate seeds of different sizes and these seeds were planted, seedlings from the smallest seed fraction demonstrated the highest rate of seed transmission. Also, when three pedigree cv. Gungurru seed crops with 1–2% seed-infected plants were rogued early to remove them and representative seed samples were tested after harvest, only 0.1–0.2% of seedlings were CMV infected. Thus, both sieving of infected seed stocks before sowing and early roguing can help reduce the number of seed-infected plants [112].

CMV contamination of the Australian national narrow-leafed lupin breeding program commenced with crosses using infected plants as parents and was subsequently carried over via seed-borne infection to each successive generation sown [112]. Seedlings of parental lines being used for crossing, F1-generation plants, early-generation breeding lines and wild germplasm were all showing symptoms typical of seed-borne seedling infection (Figure 3C–E). Aphid vectors then spread the virus first to nearby plants within the same single-row plot (Figure 3F–H) and afterward to other rows (Figure 3I), resulting in widespread infection. In addition, CMV spread also occurred from seed-infected volunteer lupin plants growing from infected seed left behind from previous years to other volunteer lupin plants causing current-season infection (Figure 3J,K). After the magnitude of the CMV outbreak in both the breeding program and newly released cultivars became evident, stringent procedures were adopted to manage CMV by creating a ‘clean seed pipeline’. From 1986 onwards, all plots of germplasm accessions, parental lines and breeding lines were rogued intensively to remove symptomatic plants with seed-borne or current-season infection. This proved very effective at decreasing the current season’s spread. From 1987 onwards, the crossing of parental lines outside was abandoned and moved to the glasshouse instead, where all symptomatic plants were removed and aphids could be controlled effectively [44,112]. CMV contamination of commercial seed stocks was also the source of the widespread epidemics occurring in WA narrow-leafed lupin crops [44], as suggested previously for the 1983 epidemic in SA [115]. These epidemics were most widespread and damaging in crops growing in the WA grainbelt’s high rainfall zones, and samples from commercial seed stocks from high rainfall zones had higher levels of seed-borne CMV than seed stocks from medium or low rainfall zones. Although seed-infected seedlings growing within sparsely growing crops survived, those within densely growing crops tended to become shaded out. Also, healthy seedlings survived better than infected seedlings under dry conditions. Thus, high plant density and drought conditions decreased the seed-borne infection sources for spread by aphid vectors, so the less frequent occurrence of CMV epidemics in low rainfall zones seemed to be due to the scarcity of aphid vectors and poor survival of CMV-infected seedlings under dry conditions [112]). From 1987 onwards, narrow-leafed lupin crops grown to produce basic or certified seed were moved to lower rainfall zones, and farmers in high rainfall zones were recommended to source seed from there. Regardless of rainfall zone, farmers were recommended to promote rapid canopy development by sowing crops at high seeding rates with narrow row spacing in order to minimize seed-infected plant survival. To avoid sowing infected seed stocks, a seed testing service was provided for farmers to get representative seed samples tested for CMV infection before sowing [44,120].

In a study in southwest WA, seed samples from primary pods, first-order pods and a combination of second- and third-order pods growing on CMV-infected cv. Illyarrie plants were tested. The rates of seed transmission found were least for primary pods (3%) and greatest for second-/third-order pods (13%), showing that seed infection was greatest in later formed pods [112]. In another WA study, when seed yield losses were examined within individual cv. Gungurru plants that became infected with CMV at different growth stages, these were least when symptoms appeared after flowering (19% loss) but greatest (56–73% losses) when symptoms first appeared prior to or during flower initiation [93]. In a study in SA, when individual cv. Illyarrie plants were inoculated with CMV at different growth stages, 45% of those inoculated as seedlings died [121]. When inoculated at the mid-vegetative growth stage, their seed yield loss was 91%, whereas it was 75% following inoculation at a late growth stage. The CMV seed transmission rates recorded depended on the plant growth stage when infection first occurred, with the highest seed transmission rate occurring after mid-vegetative growth stage inoculation (25%). The greater seed yield losses and harvested seed infection in this SA study may reflect the use of severe CMV isolate (BSA) originally from faba bean [122,123]. By contrast, the two WA studies were with natural CMV infections, so they did not involve artificial inoculation with a single isolate [93,112]. These findings suggested that, when sowing an infected seed stock, delaying CMV spread by minimizing the proportion of seed-infected plants is likely to diminish both seed yield losses and infection of harvested seed.

#### 3.1.2. Alternative Hosts

In SA in the early 1980s, tests on leaf samples from plants of other species associated with CMV-infected narrow-leafed lupin crops found CMV infecting plants of subterranean clover, burr medic (*Medicago polymorpha*), capeweed (*Arctotheca calendula*) and the weed *Erodium* sp. [115]. In an SA study published in 1992, the responses of seven narrow-leafed lupin cultivars to inoculation with 16 CMV isolates from diverse host species were recorded [122]. All lupin cultivars became infected systemically by most isolates but differed in which isolates failed to cause infection. Symptoms varied in severity depending upon the isolate and cultivar inoculated, e.g., cv. Illyarrie plants were either soon killed (five isolates), developed symptoms without necrosis (seven isolates) or developed asymptomatic infection (four isolates). In southwest WA in the early 1980s, tests on samples from plant species associated with CMV-infected narrow-leafed lupin found it infecting subterranean clover and murex medic (*M. murex*), the wild clovers hare’s foot clover (*T. arvense*) and hop clover (*T. campestre*), and the weed hosts capeweed, wild radish (*Raphanus raphinistrum*), fumitory (*Fumaria officinalis*), stagger weed (*Stachys arvensis*), King Island melilot (*Melilotus indica*), spreading stonecrop (*Crassula decumbens*), flatweed (*Hypochaeris glabra*), hyssop loosestrife (*Lythrum hyssopifolia*), lesser snapdragon (*Misopates orontium*), *Monopsis simplex* (no common name) and cornspurry (*Spergula arvensis*) [112].

During 1987–1993 in southwest WA, an extensive study of alternative hosts associated with CMV infection of narrow-leafed lupin crops and subterranean clover pastures, and of experimental plots of both species, included manual inoculations, field surveys, field experiments where virus spread was by naturally occurring aphid vectors, and tests for seed transmission [124]. Eight alternative host species were frequently found infected in the field, and another nine species became infected occasionally. The eight species infected most often were the pasture legume murex medic, the wild clovers hare’s foot clover, drooping flowered clover (*T. cernuum*), yellow suckling clover (*T. dubium*) and clustered clover (*T. glomeratum*), and the weeds spreading stonecrop, King Island melilot, and wall fumitory. Low rates of CMV seed transmission to seedlings (<1%) were detected in burr medic, spreading stonecrop and King Island melilot but not in any other of these alternative hosts [124]. By contrast, other WA studies with pasture legumes reported seed transmission of CMV to seedlings in subterranean clover (up to 9%) [125,126] and in both burr (up to 13%) and murex medics (up to 2%) [127]. However, CMV survived poorly over the dry summer period in grazed subterranean clover swards, with traces of infection through seed transmission never persisting more than five years after sowing [124]. Indeed, McKirdy and Jones [124] concluded that, under broadacre agriculture conditions in the Mediterranean-type climate southwest WA grainbelt, infected alternative hosts are unlikely to play a significant role as sources of CMV infection for narrow-leafed lupin crops sown with healthy seed. This conclusion was made because (i) although relatively high rates of seed transmission sometimes occur in the pasture legumes subterranean clover, burr medic and murex medic, CMV persisted poorly in grazed swards sown with infected subterranean clover seed and pastures sown with the two medic species rarely occur in the grainbelt region; and (ii) survival through the dry summer period in dormant weed seed requires seed transmission, but this was only found at low levels (<1%) in two weed species, one of which is rarely found in this region (King Island melilot), and was absent in any of the wild clover host species tested. Therefore, because CMV is so readily seed-borne in narrow-leafed lupin, these studies suggested that seed-infected narrow-leafed lupin plants within the crop were the primary virus source plants for its spread by aphid vectors to other lupin plants [92,93,99,112]. Because of SA’s similar climate, the scenario is likely to be similar in its SA grainbelt, but alternative CMV hosts could play a greater role elsewhere in Australia where narrow-leafed lupin is grown, and rainfall in summer allows alternative hosts to persist all year round (e.g., NSW) [34,128].

#### 3.1.3. Cultural and Phytosanitary Control

**Figure 4:**
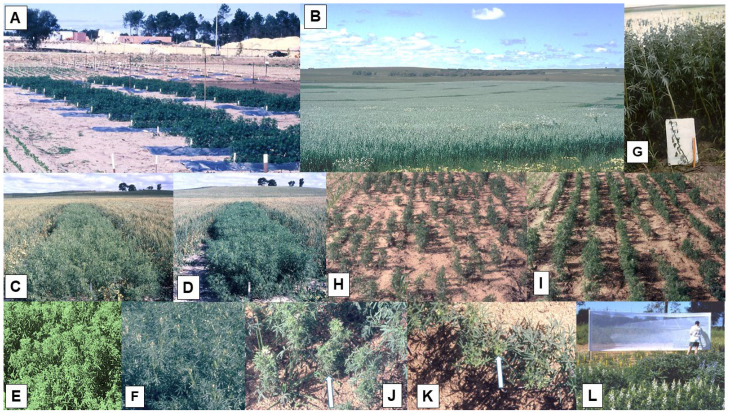
Field experiments examining the effectiveness of different control measures at controlling cucumber mosaic virus (CMV) spread in narrow-leafed lupin stands (**A**–**K**) and aphid transmission by different aphid vector species in the field (**L**). (**A**), Arrangement of single-row plots of narrow-leafed lupin with or without reflective mulch in a field experiment (South Perth 1987). (**B**), Typical arrangement of multi-row plots of narrow-leafed lupin separated from one another by wide (30 m width) non-host (cereal) buffers in field experiments examining the use of different cultural, phytosanitary or chemical control measures to suppress CMV spread (Badgingarra 1995). (**C**) and (**D**), Lupin plots at the end of the growing season, which were originally sown with 5% CMV-infected (**C**) or healthy (**D**) seed showing chlorotic foliage symptoms resulting from comprehensive current-season CMV infection (**C**) or remaining healthy due to lack of CMV spread (**D**) (Badgingarra 1989). (**E**) and (**F**), Late growing season images of portions of plots originally sown with 5% infected (**E**) or healthy (**F**) lupin seed showing current-season CMV foliage symptoms in all plants (**E**) or remaining healthy (**F**) (Badgingarra 1989). (**G**). Commercial lupin crop sown at high seeding rate showing shading out of the dwarfed seed-infected plant by a dense canopy of healthy lupin plants (Three Springs, 1988) (**G** image credit: Department of Primary Industries and Regional Development @ Peter Nelson). (**H**) and (**I**), Plots recently sown at a low seeding rate with lupin seed at narrow (**H**) or wide row (**I**) row spacing (Badgingarra 1992). Images (**J**) and (**K**) both show single seed-infected plants (indicated by white arrows) growing within narrow- (**J**) or wide-spaced (**K**) rows; the greater crowding of plants within wide-spaced than narrow-spaced rows is evident (both images also show plants with current-season CMV symptoms, which differ from seed-infected plants by the normal appearance of their lower leaves) (Badgingarra 1992). (**L**), Fine net used to trap and remove airborne winged vector aphids downwind of CMV-infected lupin plants (South Perth 1996). Figure (**L**) is modified from Berlandier et al. [130].

In the early phases of lupin breeding programs, single-row plots are widely used, where many crosses, breeding lines and accessions are evaluated. CMV spreads readily among them (Section 3.1.1 above). From 1987 to 1989 in WA, field experiments studied whether surrounding single-row plots of narrow-leafed lupin with reflective aluminium painted polythene mulch would help repel aphid landings, thereby decreasing CMV spread [129]. In 1987, cv. Illyarrie healthy seed or seed with 2% CMV infection was sown in plots, which were either left unprotected or protected by mulch (Figure 4A). Within the plots sown with infected seed, aphid vectors caused the current-season spread of CMV. This reached 30% of plants without protection but only 11% of protected plants. Moreover, vector aphids also spread CMV at a lower level from these plots to unprotected plots sown with healthy seeds (12% infection). In contrast, no CMV spread occurred in mulch-protected plots sown with healthy seeds. In 1988 and 1989, healthy cv. Danja seed was sown, and the protected and unprotected plots were alternated so the same treatments were never side-by-side. In 1988, the few infections that occurred were limited to one unprotected plot. This was because a substantial primary CMV infection source was absent from the experimental site. In 1989, vector aphids spread CMV from a nearby virus resistance screening experiment with narrow-leafed lupin (Section 3.1.5 below) to both types of plots, and this current-season spread was slower to protected plots than unprotected plots. Then, in the protected plots, after the initial development of infection foci within individual plots, aphids spread it along the rows to fewer plants. The final incidences of current-season CMV infection within unprotected and protected plots were 57% and 8%, respectively. *A. kondoi* and *M. persicae* were the only CMV vector species found infesting legumes (including lupins) at the site used from 1987 to 1989 [129]. These field experiments showed that deploying reflective mulch to protect single-row plots in the early phase of a narrow-leafed lupin breeding program will help diminish CMV spread. Therefore, this practice was recommended to complement roguing of seed-infected source plants (Section 3.1.1 above).

In 1987, when there was a 3-week drought after sowing, three field experiments with CMV in narrow-leafed lupins focused on studying the survival of seed-infected plants in plots sown with 1, 2 or 10% infected cv. Illyarrie seeds. Only 20% of seed-infected plants survived at the two drought-affected sites, but at the single irrigated site, 95% of them survived [112]. In 1989, CMV-infected cv. Gungurru seed was sown at three different depths in a field experiment. In plots with 8 and 11 cm sowing depths, the proportion of seed-infected plants was c.15% and c.50% smaller, respectively, than it was in plots with 5 cm sowing depths [92]. Thus, greater soil moisture favours their survival, but deep sowing reduces it. In four field experiments in 1998 and 1999 (two per year), lupin seed with 5% or 0.5% CMV infection and healthy seed was sown in widely separated plots. The experimental plot layout used resembled that shown in Figure 4B. Naturally occurring aphids spread the virus from seed-infected to healthy plants within the plots sown with infected seed [92]. In three experiments, in plots sown with 5% infected seed, the resulting CMV spread caused seed yield losses of 34–53%, individual seed weight was diminished by 13%, and infection in harvested seed reached 6–13% (Figure 4C–F). There was insufficient CMV spread to cause statistically significant yield losses in plots sown with 0.5%-infected seed despite 1–7% CMV infection being reached in their harvested seed. In the fourth experiment, which was in 1999 and was severely drought affected, both CMV spread and seed yield were considerably smaller. In plots sown with 5% or 1% infected seed, current-season CMV symptoms only reached 6% and 1% of lupin plants at the last growth stage such assessments were possible (drought symptoms prevented later assessments), and CMV infection in harvested seed only reached 1.6% and 0.1%, respectively. Therefore, there was no significant effect of infection on the seed yield of plots sown with 5% or 0.5% infected seed and the infection level in seed harvested was 3–5 times less than that in sown seed.

From 1990 to 1992, six field experiments were conducted with lupin seed with 0.5–5% CMV infection or healthy seed [93]. The percentage of seed-infected plants becoming established differed between experiments: 48% (exp 4), 38% (exp 1), 27% (exp 2), 24% (exp 3), 1% (exp 5) and 13% (exp 6). This was because higher soil moisture levels after sowing favoured seed-infected plant survival and establishment. When seed with 5% infection was sown in 1990, the CMV spread based on plants with current-season symptoms last recorded was 46% (exp 1) and 38% (exp 2) (but it continued to spread further subsequently). This virus spread was sufficient to diminish yield losses by 35% (exp 1) or 27% (exp 2) and cause 15% (exp 1) or 9% (exp 2) infection in harvested seed. The corresponding figures for the last recorded current-season spread from sowing 1% infected seed were 7% (exp 1) and 8% (exp 2). The spread that occurred afterward was sufficient to cause a statistically significant yield loss in exp 1 (16%) and also resulted in 5% (exp 1) or 4% (exp 2) infection in harvested seed. In two of the three 1991 experiments (exps 3 and 4), aphids arrived late, resulting in little CMV spread, therefore sowing seed with 0.5–5% initial infection did not cause significant yield losses, and there was significantly smaller CMV infection in harvested seed than in the seed sown (only 0.1–0.8%). In the third 1991 experiment (exp 5) and the 1992 experiment (exp 6) at the same site, the current-season CMV spread that occurred when seed with 3% or 5% infection was sown diminished seed yields by 25–27% (exp 5) and 37–42% (exp 6), and in exp 6 the infection levels in harvested seed resembled those in sown seed (4–6%) (seed from exp 5 not tested). Sowing 0.5, 0.75 and 1% infected seed caused statistically significant yield losses of 16–19% in exp 6, but in exp 5, there were no statistically significant yield losses from sowing 0.5, 0.75, 1 and 2% seed. Furthermore, in exp 6, the harvested seed had similar infection levels (0.4–2%) to the seed sown. The differences in outcomes between these two experiments reflected the greater aphid vector activity and consequent current-season CMV spread that occurred in exp 6 than in exp 5.

The findings from the 12 field experiments summarised in the previous two paragraphs showed that the level of CMV infection in sown seed constitutes a critical determinant of the extent of virus spread that develops, which, in turn, determines the magnitude of the seed yield losses and infection of harvested seed. They also showed that climatic conditions are a critical factor for CMV spread because they influence both the survival of seed-infected plants and aphid vector activity. This set of field data formed the basis of recommendations on acceptable threshold levels of infection in lupin seed based on testing representative samples from farmer’s seed stocks for CMV prior to sowing time. These recommendations were to only sow seed stocks with <0.1% seed infection in high rainfall zones (highest risk) or with <0.5% seed infection in medium and low rainfall zones (lower risk) [44,61,93,131]. The <0.5% threshold level for lower-risk areas was adequate to avert significant seed yield losses except in very unusual situations of exceptional pre-growing season rainfall. By contrast, the <0.1% seed threshold level (equivalent to a 1000 seed sample negative test) was necessary to avoid the more likely risk of significant yield losses in higher-risk areas. To avoid the need for growing out seedlings for routine serological testing of lupin seed samples for commercial or regulatory purposes, a polymerase chain reaction (RT-PCR) procedure for testing ground, dry seed samples was developed [132]. This provided a more sensitive, reliable and labor-saving test that detected levels of CMV seed infection as low as 0.1% reliably. It was widely adopted as a routine dry seed test in which bulked subsamples from ungerminated lupin seed were ground to a fine powder for testing, providing an estimate of the percentage of CMV seed transmission to seedlings. In a subsequent study, although it was detected in the embryo of lupin seed, CMV contamination of the seed coat was shown to be absent, validating this diagnostic approach that can advise growers on whether seed stocks are suitable for sowing [133,134].

In 1990 and 1991, four field experiments (2 per year) examined the effect of lupin plant density on seed-infected plant survival and current-season CMV spread [93]. To provide a range of plant densities, cv. Gungurru seed with 10–15% CMV infection was sown at seeding rates of 20–150 kg/ha. The seeding rates used were 20, 40, 60, 80 and 140 kg/ha in 1990, and 25, 50, 75, 100, 125 and 150 kg/ha in 1991, and as expected, plant density increased proportionally with the seedling rate in each field experiment. In exps 1, 3 and 4, survival of seed-infected plants prior to canopy closure declined with increased plant density and was always greatest at the lowest plant density generated by the lowest seeding rate. However, this effect was only statistically significant in exp 1, in which survival at the highest plant density was significantly lower than survival at the lowest plant density. In these three experiments, complete canopy closure occurred at the highest plant densities, partial closure at immediate plant densities and no closure at the lowest plant densities. There were significant reductions in percentage seed-infected plant survival due to canopy formation in all three experiments: from lowest to highest plant densities, these survival percentages ranged from 10 to 0.1% (exp 1), 2.5 to 0.2% (exp 2) and 3.4 to 0% (exp 4). Meaningful data on seed-infected plant survival could not be collected from exp 2. This was because it was sown much earlier than exps 1, 3 and 4, and aphid vectors arrived much earlier, resulting in current-season CMV spread well before canopy formation, which never became complete. In all four experiments, the percentage of plants with current-season CMV infection was greatest at the lowest plant density and least at the highest plant density. However, this trend was confirmed to be statistically significant only in exp 4; in exps 1–3, the data could not be analysed statistically as they were collected for the overall treatments but not from each replicate plot. Therefore, lower survival of seed-infected plants was associated with higher plant density and more complete and earlier canopy formation. This was due to shading out of stunted CMV-infected plants (Figure 4G). However, if aphids arrived prior to canopy formation, regardless of plant density, significant CMV spread still resulted. These findings supported the recommendation to promote canopy cover by sowing early at high seeding rates with the objective of maximising early death of seed-infected plants so they are no longer a source for current-season spread [93].

In 1992, a field experiment in which 15% CMV-infected cv. Gungurru seed was sown studied the effects of two different row spacings (17.5 and 35 cm) and three different seeding rates (60, 80 and 100 kg/ha) upon the survival of seed-infected plants (Figure 4H,I) [95] Significantly greater numbers of seed-infected plants survived with narrow than wide row spacing, their overall average survival decreasing from 79% (narrow spacing) to 43% (wide spacing). The poorer survival of seed-infected plants within rows with wider spacing rather than narrower spacing resulted from the doubling of plant numbers within each row, causing greater competition between neighbouring seed-infected and healthy plants and shading out of infected plants (Figure 4J,K). However, there was no significant effect of the seeding rate on seed-infected plant survival. In 1992–1993, in two field experiments, either 5% (exp 1, in 1992) or 10% (exp 2, in 1993) CMV-infected cv. Gungurru seed was sown at wide row spacing (35 cm). Two different seeding rates (30 and 60 kg/ha in exp 1; 40 and 80 kg/ha in exp 2) and top-dressing superphosphate fertiliser versus banding it below the sown seed (150 kg/ha in exp 1; 157 kg/ha in exp 2), were used. It was hypothesised that the boost in early growth granted from banding superphosphate and, thus, increasing available phosphate would result in an increase in the survival of seed-infected plants. As expected, there was a significant effect of seeding rate upon seed-infected plant survival, with fewer seed-infected plants persisting at the high seeding rate. However, although their survival at the higher seeding rate increased from 47% with banding to 73% with top dressing, overall, there was no statistically significant effect of superphosphate placement. Again, this study suggested that greater competition between neighboring plants results in a higher rate of shading of less vigorous seed-infected plants by healthier plants [95].

One field experiment in 1992 and four in 1995 studied the effects of cereal straw groundcover in deterring aphid vectors from landing in lupin cv. Gungurru stands and spreading CMV from seed-infected to healthy plants [95]. The level of CMV infection in the seed sown was 5% (exp 1 in 1992) or 7% (exps 2–4 in 1995). In exp 1, seed was sown at 40 and 80 kg/ha in wide row spacings (35 cm), and straw groundcover was applied to half the plots of each type at 2 kg/ha. The presence of straw significantly decreased current-season CMV spread but not the percentage of seed-infected plants that survived, and there were no significant effects attributable to the seeding rate. In exps 2 and 3, seed was sown at 100 kg/ha in wide row spacing (35 cm), and the rates of straw application were 0, 1, 2 and 4 kg/ha. A single treatment with narrow row spacing (17.5 cm) and without straw groundcover was included. There were no significant effects on seed-infected plant survival in either experiment. However, there was a significant interaction between the rate of straw application and row spacing; CMV spread was greatest at narrow spacing without straw, intermediate at wide row spacing with 0, 1 and 2 kg/ha of straw and at least at 4 kg/ha of straw. In exp 4, wide row spacing (35 cm) and a seeding rate of 70 kg/ha were used, and the plots were sown with either tynes or discs and either with or without straw (2 kg/ha). As in the previous experiments, straw groundcover significantly suppressed current-season CMV spread. However, both the survival of seed-infected plants and current-season CMV spread were significantly reduced when tynes were used rather than discs to sow the plots. This can be explained by the greater irregularity of sowing depth, straw burial and soil disturbance that occurred when discs were used. In summary, these experiments show that straw groundcover at 2 kg/ha can reduce aphid vector landing rates and subsequent current-season CMV spread and that this effect was enhanced using 4 kg/ha straw. Furthermore, exp 4 demonstrated that minimum tillage using tynes to sow CMV-infected lupin seed could also help to diminish current-season CMV spread [95].

In the field experiments described above in this section, three aphid species colonised narrow-leafed lupin plants. These were *M. persicae* (Figure 2A)*, Acyrthosiphon kondoi* and *A. craccivora* [92,93,95,112,129]. Two non-lupin-colonizing aphid species were sometimes found in large numbers colonising the cereal barriers that separated them: *Rhopalosiphum padi* (the oat aphid) or, less often, *R. maidis* (the corn-leaf aphid) [93,95]. Non-lupin-colonising aphid species trapped flying above the lupin field experiments included the non-lupin-colonising species *R. padi*, *R. maidis*, *Lipaphis pseudobrassicae* (the turnip aphid) and *Brachycaudus rumexicolens* (the dock aphid), and the three lupin-colonising species, *M. persicae*, *Acyrthosiphon kondoi* and *A. craccivora*. The species composition, timing and abundance of the flying aphids caught differed by site and year, but *L. pseudobrassicae* and *R. padi* were normally the most numerous and *M. persicae* and *A. craccivora* the least numerous [93,95]. When acquisition access times of 5–10 min were used, the aphid CMV transmission efficiencies from lupin-to-lupin found were 10.8%, 9.4%, 6.1% and 3.9% for *M. persicae*, *A. kondoi*, *A. craccivora* and *L. pseudobrassicae*, respectively [92]. From 1993 to 1996, the roles of five additional non-colonising aphid species as CMV vectors were investigated. With 5–10 min acquisition access times, the lupin-to-lupin transmission efficiencies of these were *R. maidis* 9%, *R. padi* 5%, *Therioaphis trifolii* (spotted alfalfa aphid), 4%, *Sitobion miscanthi* (the Indian grain aphid) 2% and *B. rumexicolens* 0.9% [130]. During 1993–1994 at a grainbelt site and in 1993 and 1996 at an irrigated urban site, vertically oriented nets were placed downwind of CMV-infected lupin stands to catch flying aphids. Each aphid was removed from the net, transferred to a lupin plant in situ (one aphid/plant), left to probe for 1 h and then preserved for subsequent species identification (Figure 4L). The numbers of aphids that transmitted CMV were 64/2833 and 12/186 for the grainbelt and irrigated sites, respectively, and the aphid species that transmitted it were *A. kondoi* *A. craccivora*, *Acyrthosiphon pisum*, *B. rumexicolens*, *L. pseudobrassicae*, *M. persicae, R. padi*, *R. insertum* (the apple-grain aphid), *T. trifolii* and *Toxoptera citricidus* (the citrus aphid). At the grainbelt site, *A. kondoi* proved the most important CMV vector, accounting for 50% of transmissions, *R. padi* for 22%, and *M. persicae* for 16%, and these species were trapped most often, making up 28%, 13% and 37% of those caught, respectively. At the irrigated site, *R. padi* was responsible for half of the CMV transmissions, *A. kondoi*, *R. padi*, *M. persicae* and *T. citricidus* being the species caught most often [130]. This study suggested that the lupin-colonising species *M. persicae* and *A. kondoi,* and non-lupin-colonising species *R. padi* and *L. pseudobrassicae* are the most important CMV vectors in lupin crops.

#### 3.1.4. Chemical Control

Insecticide application to kill aphid vectors and thereby reduce CMV spread might provide an additional low-cost control measure suitable for use in combination with phytosanitary, cultural and host resistance approaches in IDM. From 1990 to 1992, four field experiments at two sites studied the effects of applying carbamate or organophosphate insecticides upon current-season CMV spread in narrow-leafed lupin [94]. The experimental plot layout used resembled that shown in Figure 4B. Seed stocks of cv. Gungurru that were healthy or 5% CMV seed-infected were sown in narrow row spacing at seeding rates of 60–80 kg/ha. In exp 1, the carbamate foliar insecticide pirimicarb and the organophosphate seed dressing disulfoton were applied alone or in combination; in exp 2, pirimor alone, the organophosphate methamidophos alone or pirimicarb + the organophosphate thiometon were applied as foliar sprays; in exp 3, foliar sprays of methamidophos were applied alone or of primicarb + thiometon in combination; and in exp 4, foliar sprays of methamidophos were applied alone. In plots sown with CMV-infected seed, current-season CMV spread was significantly smaller when either pirimicarb (1/1 exp) or methamidophos (3/3 exps) were applied every 2 weeks to foliage and when disulfoton seed dressing was combined with two weekly foliar pirimicarb applications (1/1 exp). Moreover, in a single experiment each, these three treatments increased seed yield significantly (by 24–35%). When methamidophos, primicarb or mixtures of pirimicarb + thiometon were applied as single or double foliar applications, they diminished current-season CMV spread significantly in some experiments but not others. Neither disulfoton seed dressing alone nor single or double foliar applications with pirimicarb alone reduced CMV spread significantly. *M. persicae*, *A. kondoi* and *A. craccivora* were the only aphid species to colonise the lupins during these experiments. Metamidophos was more effective than pirimicarb at decreasing their numbers. Also, primicarb proved less effective in reducing colonising populations of *M. persicae* than of *A. kondoi* and *A. craccivora.* In addition to these three species, the non-colonising aphids caught on sticky traps located above the experiments were *R. maidis*, *R. padi*, *B. rumexicolens* and *L. pseudobrassicae* [94]. In summary, insecticide application to commercial lupin crops every 2 weeks is neither economically viable nor ecologically desirable despite such applications of methamidophos and pirimicarb being effective for decreasing current-season CMV spread. In addition, although single or double foliar insecticide applications of methamidophos or primicarb + thiometon sometimes diminished CMV spread significantly, this did not occur consistently, so they could not be recommended for commercial use [94].

**Figure 5:**
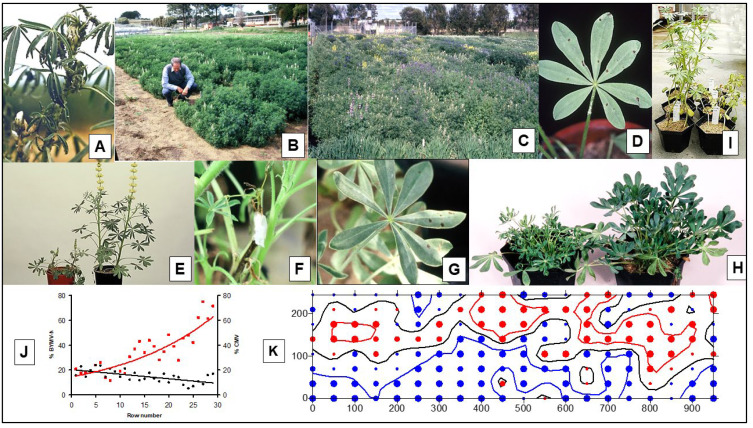
Direct aphid feeding damage, host resistance to cucumber mosaic (CMV) virus and spatial spread patterns of CMV-infected lupin plants. (**A**), Direct aphid feeding damage caused by colonisation of a narrow-leafed lupin plant by *Myzus persicae*. (**B**), CMV screening procedure involving single-row plots employed every year to evaluate wild germplasm accessions, early-stage breeding lines and/or cultivars of narrow-leafed lupin for CMV resistance: A uniform CMV inoculum source exposure was achieved by separating adjacent plots using ‘spreader rows’ planted with cv. Wandoo seed with a high CMV seed transmission rate and naturally occurring aphid vectors spread CMV from seed-infected plants to healthy plants. Irregular plant heights visible within spreader and test rows reflect plant infection at different plant growth stages (South Perth 1997). (**C**), Same single-row plot CMV screening procedure with alternate spreader and test rows being used to evaluate wild germplasm accessions, breeding lines and/or cultivars of diverse lupin species for CMV resistance (South Perth 1993). (**D**), Sap-inoculated leaf of yellow lupin cv. Motiv showing a localized hypersensitve resisistance (LHR) response consisting of necrotic local lesions. (**E**), Plants of yellow lupin cvs Motiv (right) and Reda (left) sap inoculated with CMV isolate SN showing a systemic susceptible phenotype involving severe plant dwarfing in Reda but complete lack of systemic invasion in Motiv resulting from its LHR inoculated leaf phenotype. (**F**), Yellow lupin cv. Popiel plant showing an LHR phenotype (necrosis localized to the stem immediately beneath the graft union) following graft inoculation with a CMV-infected scion. (**G**), *L. hispanicus* P26858 leaf showing an LHR phenotype (necrotic local lesions) after sap inoculation with CMV. (**H**), *L. hispanicus* P26858 (right) and P26853 (left) plants showing an LHR phenotype preventing any systemic invasion or a susceptible phenotype (leaflet downcurling, mosaic and size reduction, and severe plant dwarfing), respectively. (**I**), Pearl lupin plants sap inoculated with CMV showing a severe susceptible phenotype (leaflet mosaic, chlorosis, deformation and size reduction, and plant dwarfing) (right) compared with healthy plants (left). (**J**), Gradients of plants with current-season symptoms of CMV or bean yellow mosaic virus necrotic strain (BYMV-N) infection across a 10.4 × 15 rectangular plot of lupins in 1994 at South Perth; incidences of plants with CMV (red line and ■) and BYMV-N (black line and ■) infection. CMV and BYMV sources were on the plot’s right and left sides, respectively, causing a steeply declining curved infection gradient typical of a polycyclic spread pattern (CMV) and a shallow linear gradient typical of a monocyclic spread pattern (BYMV-N). (**K**), Clustering index (v) map of plant numbers with current-season CMV symptoms within a rectangular 2.8 m × 10 m lupin plot sown with 7% CMV-infected seed in 1996 at Badgingarra. The map shows the predominantly localized CMV spread around seed-infected infection foci. Distances shown are in cm. Spots denote quadrat units consisting of infection patches with v > 0 (red) or gaps with v < 0 (blue). The v values represented by spots of different sizes are large spots > 1.5 or <−1.5, intermediate-sized spots of +/−1 to +/−1.49 and small spots of 0 to +/−0.99. Black lines are zero-value contours, red lines around patch clusters are v = 1.5 contours and blue lines around gap clusters are of v = −1.5 and represent patch and gap regions cluster boundaries with counts close to the sample mean. (**C**,**D**,**H**) are modified from Jones [51]. (**J**,**K**) are modified from Jones [135].

From 1995 to 1997, five additional field experiments using five insecticides belonging to organophosphate, thiophosphate, pyrethroid or neonicotinoid insecticide groups were performed with CMV in narrow-leafed lupin at three locations [96]. Healthy or 7% CMV seed-infected cv. Gungurru seed stocks were sown in narrow row spacing at a seeding rate 80 kg/ha. This study compared the effects upon CMV spread of foliar application with methamidophos (organophosphate), the most effective insecticide used by Bwye et al. [94], triazamate (thiophosphate), alphacypermethrin (pyrethroid) and imidacloprid (neonicotinoid). These insecticides were applied either singly or twice. When applied to plots sown with CMV-infected seed, alphacypermethrin and imidacloprid were sprayed at two different insecticide application rates, whereas only one rate of triazamate and methamidophos was applied. Both one and two 25 g a.i./ha foliar sprays of alphacypermethrin significantly reduced current-season CMV spread (by up to 62%), but at 12.5 g a.i./ha were less effective [96]. In contrast with the earlier findings [94], methamidophos failed to diminish current-season CMV spread significantly, as did triazamate and imidacloprid. When applied at 25 g a.i./ha to plots sown with infected seed, alphacypermethrin increased seed yields by up to 60%. As yield was unaffected by direct aphid feeding damage (Figure 5A), this increase was entirely due to suppression of CMV spread by vector aphids. However, alphacypermethrin could not be recommended for large-scale use in commercial lupin crops as the amount of CMV control obtained varied too much between different experiments. The effectiveness of alphacypermethrin, but not the other insecticides, in suppressing CMV spread was explained by the unique characteristics of pyrethroids (rapid knock down and anti-feedant activity) that can prevent probing and transmission of this type of virus (non-persistent) by both colonising (e.g., *M. persicae*) and non-colonising aphids (e.g., *R. padi* colonising the cereal barrier between plots in 3/5 of the experiments). The number of colonising *A. kondoi* and *A. craccivora* was most effectively controlled by alphacypermethrin, methamidophos and triazamate, whereas the number of colonising *M. persicae* was controlled most effectively by imidacloprid. This was explained by the fact that 85% and 82% of the colonising *M. persicae,* respectively, were found to have intermediate or high levels of E4/F4 esterase amplification, which confers resistance to organophosphates, pyrethroids and carbamates. However, whether *M. persicae* with insecticide resistance is present or absent may be of minor importance in relation to CMV transmission, especially when non-colonising aphid vector species are common [96].

#### 3.1.5. Host Resistance

From 1987 to 1993, at two locations in WA, seven field experiments were performed (1/year) to screen for CMV resistance in cultivars, breeding lines and germplasm accessions of narrow-leafed lupin [136,137,138]. Irrigation was used to optimise growth and extend the growing period to ensure that the latest flowering genotypes produced sufficient seed. Exps 1 and 2 developed and validated the ‘spreader row’, single-row plot and CMV screening procedure prior to its adoption. Uniform test row infection was obtained, along with reproducible seed transmission data, so the procedure was suitable for use for large-scale CMV resistance evaluation (Figure 5B). Exps 3–7 evaluated 57–60 test lines each. For this, spreader rows of a c.30% CMV-infected cv. Wandoo seed stock was sown on both sides of each test row, each of which was replicated twice, and naturally occurring aphids spread the virus from seed-infected plants to healthy plants within each spreader row and from there to the plants in adjacent rows. Five control lines with previously established widely differing levels of seed transmission were included. Seed harvested from each individual test row was harvested, and seedlings grown from it were tested to establish the CMV seed transmission rate to lupin seedlings. During 1989–1993, the test rows included: (i) wild accessions from the Australian Lupin Collection (exp 3); Australian parental lines used in breeding (exp 4); (ii) a mixture of introduced European cultivars Australian parental lines, advanced breeding lines, wild accessions and F4 cross progenies (exp 5); (iii) 24 artificial mutants produced by treatment with ethyl methyl sulfonate and second-generation cross progenies (exp 6); and (iv) a combination of wild accessions and advanced breeding lines (exp 7). Extreme CMV resistance was never detected in the genotypes tested. Whether partial resistance to infection was present was not established as all plants became infected because of the high CMV inoculum pressure arising from aphid vector transmission from the adjacent spreader rows. In addition, no attempt was made to see whether virus titre varied between genotypes. However, as current-season CMV symptoms ranged from severe to mild, genotype sensitivity differed. By contrast, when harvested seed from each test row was tested, differences between different genotypes were consistent for seed transmission rates to seedlings and often highly significant. The intrinsic seed transmission rates to seedlings for each genotype tested were classified as follows (% seed transmission in parentheses): moderately resistant (1–6%), moderately susceptible (6–20%), susceptible (20–35%), very susceptible (35–75%), but none were immune (0%) or highly resistant (<1%). The partial seed transmission resistance found was quantitatively inherited and under polygenic control. Evidence of this was provided by the transgressive segregation for improved seed transmission found in the progeny of certain crosses, e.g., breeding line 84L:477 had greater seed transmission resistance than either of its parents, breeding line CE2-1-1 and cv. Gungurru [137]. By contrast, flowering time and alkaloid content had no relationship to it [137]. Breeding for moderate seed transmission resistance commenced immediately after this study (in 1994), enabling new lupin cultivars with low intrinsic seed transmission rates to be released [44,61,138] and the discarding of genotypes in the very susceptible and susceptible categories. Moreover, the same CMV resistance screening procedure developed in 1987–1988 is still in use 36 years later (i.e., in 2023) [51].

In 1993–1995 in WA, three of the CMV resistance field screening experiments described for narrow-leaded lupin in the previous paragraph (Figure 5C) and graft inoculation in the glasshouse were used to evaluate several genotypes of other lupin species for their CMV resistance/host status [51,139,140]. White lupin and six different ‘rough-seeded’ lupin species (*L. atlanticus*, *L. cosentinii*, *L. digitatus*, *L. palestinus*, *L. pilosus*, *L. princeii*) never became infected with CMV when exposed to field infection from spreader rows and/or graft inoculation. By contrast, four pearl lupin, 17 yellow lupin (Figure 2D,E), and two *L. hispanicus* genotypes became infected with CMV despite another *L. hispanicus* (P26858) and seven yellow lupin genotypes remaining uninfected. In tests on seed samples from the CMV-infected yellow lupin genotypes, seed transmission rates to seedlings were 0.2–16%. These differences in seed transmission proved stable between the same genotypes across different years, so partial resistance to CMV seed transmission similar to that found in narrow-leafed lupin was suggested to be present. By contrast, in pearl lupin and *L. hispanicus*, CMV seed transmission was not detected.

Infective sap of CMV subgroup II isolate SN was inoculated to 14 yellow lupin genotypes, and 10 of these were graft inoculated with infected scions. The sap inoculation elicited a localized hypersensitive resistance (LHR) phenotype in inoculated leaves of five genotypes (Figure 5D), localisation of infection mostly without LHR in one of them (P26815) and a mixture of LHR and susceptible phenotypes in three of them. The other five genotypes developed a susceptible phenotype (Figure 5E). (Note: CMV subgroups I and II differ by <70% in nucleotide sequence identity, and subgroup II is mostly restricted to regions with temperate climates and is the subgroup found infecting cool-season pulses in Australia [102,140,141]). Graft inoculation with CMV isolate SN to the six genotypes that only developed an LHR phenotype elicited an LHR phenotype in them (Figure 5F). Graft inoculation of the three genotypes that developed a mixture of LHR and susceptible phenotypes following sap inoculation elicited a mixture of this phenotype and a susceptible phenotype in two of them but an entirely susceptible phenotype in one of the latter, demonstrating that these three genotypes lacked genetic uniformity. The LHR phenotype was slowest to spread systemically in P26815, so this was the most resistant yellow lupin genotype. Moreover, when plants of the same genotypes that developed LHR were sap inoculated with six other CMV subgroup II isolates (all from lupin) or one subgroup I isolate from banana, they always elicited an LHR phenotype. By contrast, a second subgroup I isolate from clover always elicited a systemic susceptibility phenotype. This suggested the presence of a strain-specific hypersensitivity gene in yellow lupin.

When the F2 progeny plants of a cross between hypersensitive and susceptible yellow lupin parents were inoculated with a subgroup II isolate SN, the 3:1 ratio (hypersensitive: susceptible) obtained was consistent with the presence of a single dominant hypersensitivity gene, which was thereafter named *Ncm-1* [140]. The Polish and Byelorussian (=Belarusian) yellow lupin cultivars or breeding lines containing *Ncm-1* (Popiel, Teo, Motiv, WTD1191), all had cv. Cyt as a parent in their crossing histories, so this could be its original source. Gene *Ncm-1* held up against all five of the subgroup II isolates from lupin it was challenged with, suggesting it has broad specificity and so is suitable for breeding CMV-resistant yellow lupin cultivars. When the three genotypes of *L. hispanicus* from the 1991 CMV resistance field screening experiment described above were sap inoculated with CMV subgroup I or II isolates, P26858 responded in the same way as gene *Ncm-1* carrying genotypes of yellow lupin (Figure 5G,H). *L. hispanicus* genotypes P26853 and P26859 only developed a hypersensitive phenotype with CMV subgroup I, so the presence of a second strain-specific hypersensitivity gene in this species was suggested [140]. Since CMV infection of white lupin and the six ‘rough-seeded’ lupin species mentioned above has never been reported overseas in the field [49] nor in Australia [51], they appear to be non-hosts. White lupin and sandplain lupin occur commonly in southern Australia, the former as a crop plant and the latter as a pasture plant and an introduced weed. They are, therefore, not potential sources for CMV spread to other pulses that CMV infects.

During 2002–2003, six pearl lupin genotypes were included in the annual narrow-leafed lupin screening experiments described above in this section. Although five of these genotypes were CMV infected (Figure 2E), genotype P26956 was not suggesting it might possess CMV resistance [142]. When six CMV isolates from diverse host species were sap inoculated to P26961 and P26956 plants, none of them infected P26956 despite multiple consecutive sap inoculations. By contrast, genotype P26961 was infected readily by three legume isolates (Figure 5I). The other three CMV isolates (two of which were not from legumes) also infected it, but multiple inoculations were needed to cause systemic infections in most or all plants. When P26961 and P26956 plants were graft inoculated with lupin isolate LW, P26956 plants all developed localized necrosis directly under the graft union, whereas all plants of P26961 developed a susceptible phenotype. These findings suggested that P26956 carries extreme CMV resistance, which is now available for breeding CMV-resistant pearl lupin cultivars [142].

Partial resistance to virus infection via aphid vectors, known as infection resistance, was found in germplasm accessions and breeding lines of narrow-leafed lupin during annual routine BYMV resistance screening activities [51]. As mentioned previously in this section, because virus inoculum pressure was too high when the CMV resistance screening procedure with single-row test plots and spreader rows was used each year, it was impossible to identify CMV infection resistance among the genotypes tested. Nevertheless, when naturally occurring infection with CMV was observed spreading in lupin breeding trials, the genotypes of narrow-leafed lupin present frequently became infected to different extents. Although different levels of seed infection between the genotypes sown was one likely cause, inherent differences in relative susceptibility and infection resistance between genotypes may also have contributed. To provide evidence of CMV infection resistance in narrow-leafed lupin, field experiments involving plots sown with healthy seeds of different genotypes, together with a uniformly distributed but smaller virus inoculum source, would be necessary [44,51].

Australian studies examined the feasibility of employing genetic engineering to establish CMV resistance using tobacco as a model system for CMV resistance in lupin [143]. The approach involved viral coat protein (CP) and defective replicase (DR) gene-mediated resistance. The transgenic tobacco plants were challenged by graft inoculation with five CMV subgroup II isolates from lupin and three subgroup I isolates from banana. Inoculation of plants with the DR gene revealed extreme resistance to subgroup I isolates. By contrast, challenging plants with the CP gene resulted in a smaller number of plants becoming infected, slower systemic spread in infected plants, partial symptom remission and diminished virus multiplication. Challenging plants with DR or CP genes with subgroup II isolates resulted in smaller numbers of plants becoming infected and isolate-dependent delayed systemic spread or symptom remission, especially when DR was present. The introduction of DR gene subgroup II isolate constructs into commercial lupin cultivars was recommended. Further Australian studies examined the effectiveness of resistance gene constructs obtained from CP, movement protein and replicase genes of a lupin-derived CMV subgroup II isolate. When *Nicotiana benthamiana* was transformed with these constructs, some of these carried extreme CMV resistance [144,145,146,147]. Transformation of narrow-leafed lupin plants with these constructs was achieved successfully [147], but the CMV resistance this provided appeared unstable in later generations derived from them. As yet, there have been no genetic modification studies using CRISPR or RNAi [87,88,89,90] to counter CMV infection of lupin.

In 2023, a global review on host resistance to viruses of lupins and its importance in lupin breeding programs recommended priorities for future research [51]. In brief, those most relevant to CMV research on lupins in Australia were as follows:

- Deploy speed breeding to expedite the incorporation of CMV resistance into new lupin cultivars.
- Develop molecular markers suitable for use in streamlining the breeding of new narrow-leafed and yellow lupin cultivars with CMV resistance. This needs to include identifying quantitative trait loci (QTLs) for use as molecular markers to speed up breeding for polygenically inherited resistance, e.g., to seed transmission.
- Establish whether CMV infection resistance suitable for use in breeding lupin cultivars with this trait is present in narrow-leafed lupin germplasm.
- Incorporate CMV resistance gene *Ncm-1* to provide CMV resistance during breeding yellow lupin cultivars for Australia.
- Employ accession P26956 as a parent to confer extreme CMV resistance when breeding new cultivars of pearl lupin suited to Australian growing conditions.
- Investigate the use of recently developed genetic modification procedures, especially genome editing and RNA silencing, to introduce stable resistance to CMV into new cultivars of yellow, narrow-leafed and pearl lupin.

#### 3.1.6. Patterns of Spread

In the series of field experiments described above in Section 3.1.3 and Section 3.1.4, the temporal dynamics of CMV spread from primary seed-infected plant sources to heathy plants growing within narrow-leafed lupin stands was studied by plotting progress curves for disease incidence (based on current-season symptoms in early studies) or virus incidence (based on leaf sample testing in later studies). These followed a sigmoid curve pattern with a slow start while the numbers of infected plants slowly increased, a rapid exponential spread phase as spread accelerated, and a final slowdown in spread when few healthy plants remained. Spread commenced shortly after the first arrival of aphid vectors, with progress curve steepness being related to seed-infected plant incidence. The spatial distribution of spread always involved initial clustering of new infections around seed-infected CMV source plants, followed by initiation of new infections further away. This pattern resulted from aphid vectors acquiring and spreading the virus from seed-infected plants to nearby healthy plants. Then, aphids increasingly acquired CMV from current-season-infected plants within these enlarging infection foci and spread it to isolated healthy plants further away. This process started new enlarging infection foci for further spread as aphid vectors visited them and carried the virus to more distantly located healthy plants.

In 1994 and 1996, studies at two WA sites examined the spatial patterns of CMV spread in narrow-leafed lupin stands in more detail [135]. In 1994, a 10.4 m × 15 m block at South Perth was sown with healthy cv. Gungurru seed. Along its southern edge, a narrow fallow strip originally sown with field pea that had subsequently died away separated it from an annual CMV resistance screening experiment with lupin (see Section 3.1.5 above). This resistance screening experiment acted as a potent CMV source from which vector aphids spread the virus to healthy lupin plants within the block. In 1996, the area assessed was an untreated 2.8 m × 10 m plot sown with 7% CMV-infected lupin cv. Gungurru seed and was located in an insecticide field experiment at Badgingarra [96]. Seed-infected plants within this plot acted as internal CMV sources for spread to healthy plants by aphid vectors. For each individual plant within each stand in both years, CMV infection status was recorded at regular intervals. In 1994, there was a steep gradient of CMV infection across the stand arising from a sharp decline in the incidence of infected plants between the edge closest to the CMV source and the edge furthest away (Figure 5J). This steep gradient was consistent with CMV’s polycyclic spread pattern. It contrasted with the much more gradual decline in the incidence of infected plants spreading in the opposite direction from a BYMV necrotic strain (BYMV-N) source on the opposite side of the block. This difference was a consequence of BYMV-N’s monocyclic spread [135]. When the Spatial Analysis by Distance IndicEs (SADIE) program [148,149] was used to analyse the individual plant data for CMV, the contour map obtained agreed with the steep CMV infection gradient. It revealed a marked contrast between areas closest to the source where patch clusters predominated and areas furthest from the source where gap clusters predominated. This spatial pattern formed because of (i) comprehensive, localized CMV spread by viruliferous aphids around initial plant infection foci produced by incoming vector aphids and (ii) greater numbers of viruliferous aphids then flying to, landing upon and infecting plants growing closer to where they had flown from instead of flying further and reaching more distant plants. In 1996, the SADIE contour map showed a pronounced separation between areas of a plot in which large patch or gap clusters predominated (Figure 5K). This spatial pattern was consistent with localized comprehensive CMV spread around initial seed-infected plant foci. At both sites, the current-season CMV-infected plants remained throughout the growing season, providing CMV infection sources for further cycles of acquisition and spread by aphids. Moreover, the spatial distributions of CMV-infected plants obtained in both years were typical of those obtained with a polycyclic pattern of virus spread by a non-persistently aphid-transmitted virus [135].

#### 3.1.7. Epidemic Drivers and Forecasting

Section 3.1.3 and Section 3.1.4 above describe the aphid vector species found associated with CMV spread within a series of field experiments. For the five most important lupin-colonising or non-lupin-colonising aphid vector species in the WA grainbelt [135], we summarised the aphid vector situation within the growing season as follows: modified quote—“*M. persicae*, *A. craccivora* and *A. kondoi* colonise lupin crops, *M. persicae* and *L. pseudobrassicae* colonise adjacent canola crops and wild radish (*R. raphanistrum*) weeds, and *R. padi* colonise adjacent cereals and grasses. In mixed species pasture dominated by subterranean clover, *A. kondoi* and *A. craccivora* colonise clovers and other legume species, *M. persicae* mainly colonise broad-leafed weeds and *R. padi* colonise grasses. All five species are involved to differing extents in virus transmission to and within lupin crops, the actual vector species scenario varying with site and year”. Section 3.1.2 above describes the Australian information on alternative CMV host species with the potential to act as sources for virus spread to lupin crops. However, there was no evidence of them playing a significant role as CMV sources for the spread of lupin crops in southern Australian grainbelt regions. Moreover, where weed host species growing within or near CMV-infected lupin crops were found to be infected, their occurrence was consistent with virus spread to them from infected lupin crop plants rather than vice-versa.

In 1998 and 1999, ‘data collection’ blocks (sometimes called ‘validation’ or ‘calibration’ blocks) of narrow-leafed lupin representing different rainfall and geographical regions of the WA grainbelt were sown with 7% CMV-infected cv. Gungurru seed at the same four sites [150,151]. From the nearest weather station, climatic data were collected on daily fluctuations in temperature, rainfall, and wind strength and direction. Biological data were also collected at each block, including (i) initial lupin plant density; (ii) date of first aphid vector arrival; (iii) increase in CMV incidence in lupin plants throughout the growing season (to generate virus infection progress curves); (iv) numbers of each aphid species colonising the lupin plants; (v) numbers of each colonising and non-colonising aphid species trapped flying; and (vi) extent of CMV infection in harvested seed. This information and similar data collected from field experiments performed over a 14-year period at six widely dispersed WA grainbelt sites (Section 3.1.3 and Section 3.1.4 above) were combined with data from both aerial aphid trapping and controlled environment and glasshouse experiments to provide a clear understanding of the factors driving the range of scenarios that unfolded at different sites in different years [80,84,152,153]. In brief, the magnitude of the CMV epidemics, the seed yield losses and the levels of CMV infection in harvested seed that arose differed widely with year, growing season, rainfall zone and geographical region. When CMV-infected seed was sown, the main drivers that dictated the end result included the extent of infection in sown seed, the degree of seed-infected seedling survival, the stage of crop development when aphid vectors first arrived, and the non-lupin-colonising or lupin-colonising aphid species present either within or temporarily visiting the crop, their activity and their abundance. Optimal seed-infected lupin seedling survival was encouraged by moist conditions, shallow sowing at low seeding rates with wide row spacing and delayed canopy closure. Lack of ground cover encouraged aphid vector landings, thereby increasing the extent of virus transmission. Weather factors such as rainfall, wind and temperature influenced the extent of virus transmission, aphid numbers and aphid behaviour. Heavy rainfall and high winds knocked aphids off plants, reducing the aphid population and resulting in virus transmission, but moist, warm conditions enabled plants to flourish, favouring aphid population growth and consequent virus transmission. In addition, cold winter conditions delayed symptom expression in seed-infected plants. This was due to reduced virus concentration that diminished their ability to act as effective virus sources for CMV spread by aphids until spring arrived, bringing warmer conditions and increased virus multiplication [44].

**Figure 6:**
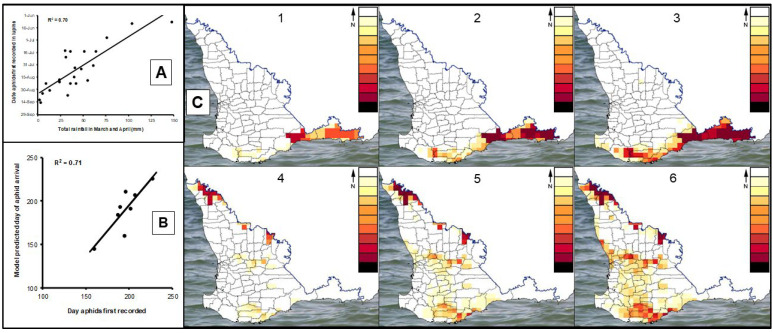
Australian forecasting model and decision support system for cucumber mosaic virus (CMV) epidemics in narrow-leafed lupin. (**A**), Relationship between the amount of annual rainfall in March and April and the first record date for aphid arrival in lupins at six different southwestern Australian grainbelt sites over the period 1988–2001. (**B**), Relationship between model predictions for the annual first record date for aphid arrival over a 2–4-year period and their actual arrival date at three locations. (**C**), CMV-lupin pathosystem risk maps for southwest Australian grainbelt in 2007 and 2008 assuming Julian day 120 crop emergence and sowing 2% CMV-infected seed. Individual forecast areas shown as square cells and shire boundaries as lines. Upper and lower rows are for 2007 and 2008, respectively: (**1**,**4**) best case (tenth percentile); (**2**,**5**) most likely (50th percentile); (**3**,**6**) worst case (90th percentile). Black to white colours represent 100% to 0–10% incidence, with 10% intervals representing colour changes between them. (**A**,**B**) are from Thackray et al. [153], and (**C**) is from Jones et al. [80].

Aphids are unable to reproduce sexually under Mediterranean-type climate conditions where rainfall is usually minimal in summer and early autumn. Over the hot, dry summer period, they persist in very low numbers upon herbaceous weed or volunteer crop host plants growing in scarce, damp locations, which occur throughout the grainbelt. These damp spots include roadside ditches where dew runoff from roads and tracks provides sufficient moisture for plants to survive, soaks where moisture reaches the surface, creek edges and irrigated gardens [44,151,153,154,155,156]. The timing and amount of rainfall in the first two months of autumn (March and April) determines the extent of aphid build-up before lupin crops are sown in mid to late autumn (April to May) and when they first arrive in growing lupin crops (Figure 6A). The consequence of having substantial early rains is that they allow a ‘green ramp’ of annual pasture, weed and volunteer crop hosts of aphids to emerge and flourish. After building up their populations on these plants, aphid vectors arrive in emerging lupin crops early (before winter). This gives rise to early CMV acquisition from seed-infected plants and initial virus spread, prolonged aphid vector activity and widespread CMV infection, which, in turn, results in greater yield losses and infection of harvested seed. By contrast, when rainfall is light and late before sowing time, the opposite outcome of minimal CMV spread, seed yield loss and harvested seed infection develops [44,151,153].

**Scheme 1:**
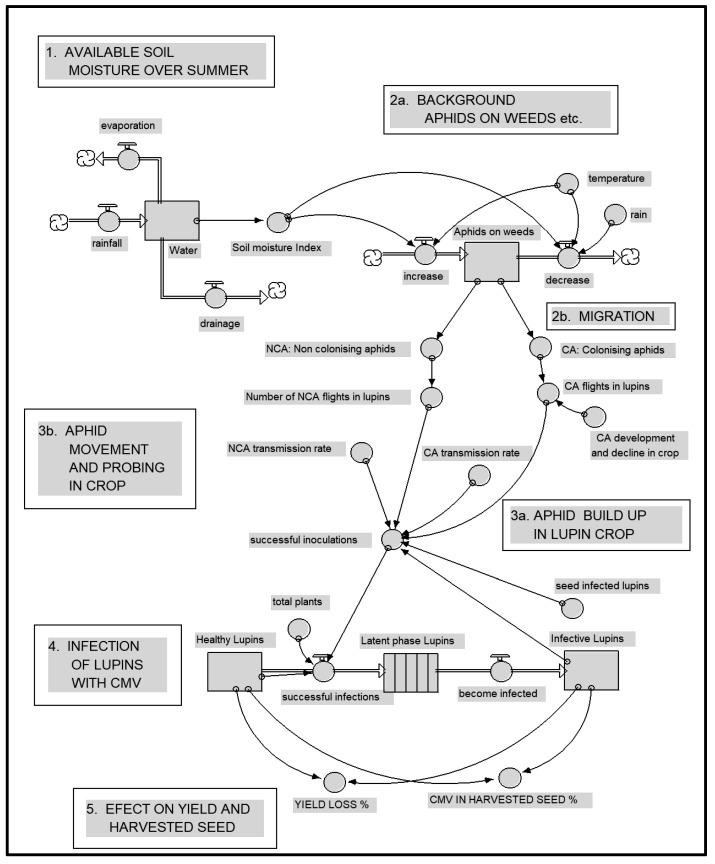
Diagrammatic overview of the cucumber mosaic virus (CMV) narrow-leafed lupin pathosystem forecasting model’s five principal components and the relationships between them. From Thackray et al. [153].

Based on the understanding of epidemic drivers summarised above, a simulation model that forecasted aphid vector activity and CMV epidemics in narrow-leafed lupin crops in the WA grainbelt was devised [80,152,153]. It employed summer and early autumn rainfall data from each location to quantify an index of aphid population increase within self-regenerating pastures, volunteer crop plants and weeds prior to the late autumn start of the growing season (Scheme 1). A forecast of the time of aphid arrival in crops was provided by the index (Figure 6B). The model then calculated aphid vector build-up within the crop, the extent of CMV current-season spread and any resulting yield losses, and the extent of harvested seed infection. Its inputs consisted of annual rainfall data, sowing date, lupin cultivar and the proportion of seed-infected plants present. It forecasted aphid vector arrival time, within-crop aphid build-up, CMV spread, lost yield and virus contamination of harvested seed (Scheme 1). Its simulations were validated by comparison with field data collected from six grainbelt sites during a 14-year period. This comparison included a diversity of rainfall scenarios prior to the growing season, sowing times, % CMV infection in sown seed and lupin plant densities. This model was combined with a DSS for use in deploying CMV control measures and insecticide applications to prevent direct aphid feeding damage. This DSS was made available via an Internet site. Subsequently, the automated DSS produced by Maling et al. [157] was employed to provide annual risk maps for CMV epidemics in lupins in different regions of the WA grainbelt [80]. For example, in 2007, the greatest risk was in southeastern coastal areas of the southwest grainbelt, whereas in 2008, it was in certain northern and central districts at the grainbelt’s eastern inland edge (Figure 6C). However, the CMV-lupin pathosystem forecasting model and DSS subsequently fell into disuse. To remedy this situation, it will need to be updated to include automated daily data retrieval from the grainbelt’s weather station network and build greater flexibility to ensure it can cope with the recent trend towards earlier sowing dates and increased climate instability now being experienced across the WA grainbelt. In addition, before its adoption by farmers in eastern Australian grainbelts, it will need adjustment to accommodate differences in their local agronomic practices and climatic conditions.

#### 3.1.8. Integrated Disease Management

In Australia, by the end of the 1990s, the CMV narrow-leafed lupin pathosystem was well understood, and the IDM strategies devised to address this major lupin crop epidemic were adopted widely in WA [10,44,62]. The IDM strategies developed for commercial lupin crops, special-purpose lupin crops and lupin breeding plots were based on the results of the 14-year research program described above. They consisted of phytosanitary, cultural, chemical and host resistance control measures, which act in different ways and target either the initial external or internal virus source or the early or later phases of virus spread. The most important component of the commercial lupin crop IDM was sowing lupin seed stocks with CMV contents below threshold levels established for higher and lower-risk zones. Its diverse components, and those for special-purpose lupin crops and lupin breeding plots, were as follows:

For commercial lupin crops:

- In grainbelt zones at higher risk of losses from CMV infection, minimize the initial internal seed-borne infection source by sowing seed stocks with <0.1% CMV infection sourced from lower-risk zones (phytosanitary).
- In grainbelt zones at lower risk of losses from CMV infection, diminish the initial internal seed-borne infection source by sowing seed stocks with <0.5% CMV infection (phytosanitary).
- Sow cultivars with intrinsic CMV seed transmission rates that are low to help minimise the initial internal seed-borne CMV infection source, especially when retaining harvested seed for sowing in the following growing next season (host resistance).
- Sow seeds at high seeding rates to generate high plant densities and early canopy closure to (i) shade out seed-infected plants and early-current-season-infected plants, thereby minimising the early internal infection source for subsequent CMV spread by aphid vectors, and (ii) diminish aphid landing rates, thereby further diminishing CMV spread (cultural).
- Sow seeds at narrow row spacing to generate early canopy closure, thereby diminishing aphid landing rates and the extent of CMV spread before canopy closure (cultural).
- When sowing untested seeds at wide row spacing in lower CMV risk zones, ensure a high seeding rate is used to produce high plant densities within rows that shade out CMV seed-infected plants, thereby reducing the primary source of inoculum (cultural).
- Sow early maturing cultivars to diminish both late CMV spread by vector aphids and additional harvested seed infection in extended growing seasons (cultural).
- Maximise stubble groundcover using minimum tillage procedures that minimise soil cultivation to diminish aphid vector landing rates, thereby reducing CMV spread prior to canopy closure (cultural).
- Employ crop rotation to avoid volunteer seed-borne lupin infection sources growing within crops (cultural).
- Ensure isolation from neighbouring pulse (including lupin) crops or legume pastures to avoid any ingress of CMV from vector aphids flying from alternative external virus sources (cultural).
- Maximise weed control using selective herbicide to minimise potential weed infection sources of CMV within the crop (chemical).
- Apply insecticides solely to manage direct aphid feeding damage once threshold population numbers are reached (chemical).

Extra items for special-purpose lupin crops:

- Sieve infected seed stocks before sowing to remove the small seed fraction before sowing, which helps reduce the number of seed-infected plants (phytosanitary).
- Mixed cropping with non-host (e.g., cereal) to diminish CMV spread to lupins gown for hay production (cultural).
- Spray high-value lupin seed crops regularly with a mixture of pyrethroid and neonicotinoid insecticides applied at high application rates to kill or repel incoming vector aphids (chemical). *

For lupin breeding sites:

- Introduce a healthy seed pipeline by keeping parental and F1-generation plants inside insect-proof glasshouses/screenhouses where any initial sources of CMV infection can be identified and removed (phytosanitary).
- Maintain a healthy seed pipeline by growing F2 and later generations in isolation from other lupins to avoid CMV re-introduction (phytosanitary).
- Discard seed lots found to be CMV-infected by testing representative seed samples from selected breeding lines outside the growing season (phytosanitary).
- Employ rigorous roguing procedures to remove plants with symptoms of seed-borne infection from plots before aphid vectors spread CMV (phytosanitary).
- Destroy CMV-infected plots with herbicide sprays or by their physical removal to avoid its spread to other plots (chemical).
- Deploy reflective mulch to protect single-row plots to decrease aphid landing rates (cultural).
- Sow plots into retained stubble or add straw mulch to decrease aphid landing rates (cultural).
- Achieve early canopy cover by sowing plots at high seeding rates with narrow row spacing to decrease aphid landing rates and shade over plants with seed-borne CMV infection (cultural).
- Sow a non-host crop perimeter around plots to act as virus ‘cleansing barriers’ against CMV ingress from external sources (cultural).
- Spray plots regularly with a mixture of pyrethroid and neonicotinoid insecticides applied at high application rates to kill or repel incoming aphids (chemical). *
- Apply selective herbicides to remove potential CMV alternative hosts from between plots (chemical).

* Note: This recommendation was first made before the extent of the negative effects of insecticides upon human health and the environment and the development of insecticide resistance in insect vectors became better understood. Whenever possible, suitable combinations of non-chemical control measures should be deployed in IDM without including insecticides to suppress insect vectors”.

The IDM strategy devised against CMV in commercial lupin crops was adopted widely in the WA grainbelt. It proved very effective, such that after its introduction up until the year 1997, this virus was rarely found causing serious disease in commercial narrow-leafed lupin crops in WA. However, complacency concerning the need for its continued adoption began to develop in the late 1990s, as evidenced by the diminishing use of the commercial CMV seed testing service [61]. This trend and the gradually diminishing attention to enforcing both the ‘healthy seed pipeline’ and seed-infected plant removal by roguing and other means within the lupin breeding program runs the risk that widespread CMV infection and consequent major yield reductions make a comeback in commercial lupin crops [44] increasing gradually thereafter. There has also been a marked trend towards the replacement of lupin cropping by canola, with the WA lupin area declining gradually from 1 million ha in 2000 to 400,000 ha in 2022 [158,159,160]. This has also contributed to the diminished attention being paid to CMV control in commercial lupin crops. Within the eastern Australian grainbelt, where narrow-leafed lupin always remained a minor crop, uptake of the CMV IDM strategy, and especially its focus on seed testing and sowing healthy seed, has been less thorough than in WA. There is a need for a vigorous extension effort focused on reminding southern grainbelt farmers and lupin breeders alike of the need for having seed samples from their lupin seed stocks tested for CMV on an annual basis and to ensure farmers apply established threshold levels for % seed infection when sowing their seed.

### 3.2. Pulses Other Than Lupins

#### 3.2.1. Occurrence in Plots, Crops and Seed Stocks

**Figure 7:**
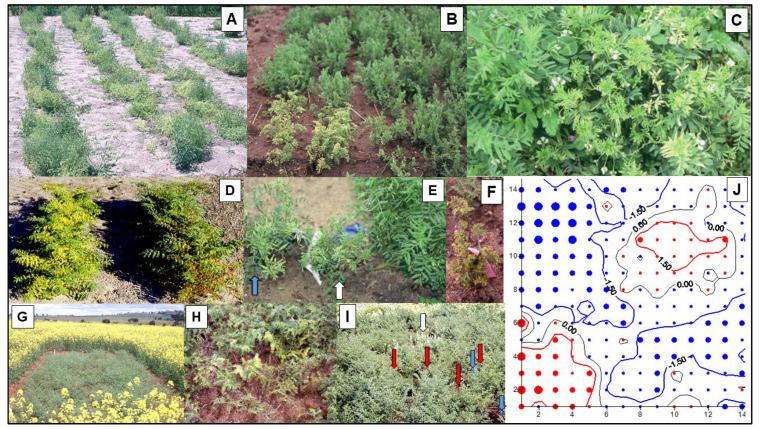
Australian field studies with cucumber mosaic virus (CMV) infecting lentil and chickpea. (**A**), Rows consisting of many plants showing foliage symptoms of leaflet chlorosis, deformation and size reduction and plant dwarfing caused by CMV infection within an experimental plot of lentil cv. Digger (South Perth 1996). (**B**), Group of plants showing foliage symptoms of leaflet chlorosis, deformation and size reduction and plant dwarfing caused by CMV infection (bottom left) growing within an otherwise healthy experimental lentil plot (Cunderdin 1997). (**C**), Plants showing foliage symptoms of leaflet chlorosis, deformation and size reduction caused by CMV (right) or lacking symptoms (left) within an experimental plot of lentil (South Perth 1997). (**D**), Rows of Kabuli (left) and Desi (right) chickpea plants showing foliage symptoms of chlorosis (Kabuli) and reddening (Desi) caused by CMV infection (South Perth 1996). (**E**), Individual seed-infected (centre) and current-season-infected (left) lentil plants that developed CMV foliage symptoms or remained healthy (right); symptoms were leaflet chlorosis, deformation and size reduction affecting all leaves (white arrow, seed infection) or without affecting lowermost leaves (blue arrow, current-season infection) (South Perth 1997). (**E**) Image credit: Department of Primary Industries and Regional Development @ Simon McKirdy]. (**F**), Infector transplant of chickpea cv. Sona in situ (pink ribbon tag) in exp 1 with leaflet symptoms of chlorosis, reddening, downcurling and bunching caused by CMV infection (Avondale 1999). (**G**), Example of an experimental plot of chickpea cv. Sona from exp 1 showing its rectangular shape (2.8 m × 10 m = 28 m^2^) and the 20 m wide non-host canola buffer that surrounded it (Avondale 1999). (**H**), Part of chickpea cv. Sona plot from exp 1 with widespread CMV infection causing foliage symptoms of leaflet chlorosis and plant dwarfing (Avondale 1999). (**I**), Part of square (20 m × 20 m = 400 m^2^) plot of chickpea cv. Tyson from exp 2 with CMV infection focus consisting of lupin plants with white flowers (white arrow) and diseased chickpea plants tagged at two different times using red tape (red arrows) or blue tape (blue arrows) (Avondale 1997). (**J**), Clustering index (v) map of plants with current-season CMV symptoms within a portion of a square 20 m × 20 m chickpea cv. Tyson plot from exp 2 with five CMV infector foci sown with infected lupin seed, one central and one near each corner (Avondale 1997). The portion of this plot shown shows the extent of CMV spread around the central and left lower corner infector foci. Distances shown are in cm. Spots denote quadrat units consisting of infection patches with v > 0 (red) or gaps with v < 0 (blue). The v values represented by spots of different sizes are large spots > 1.5 or <−1.5, intermediate-sized spots of +/−1 to +/−1.49 and small spots of 0 to +/−0.99. Black lines are zero-value contours, red lines around patch clusters are v = 1.5 contours and blue lines around gap clusters are of v = −1.5 and represent patch and gap regions cluster boundaries with counts close to the sample mean. Images (**F**) and (**H**–**J**) are from modified Jones et al. [97].

The foliage disease symptoms CMV elicits in plants of cool-season pulses other than lupins include leaflet mosaic, chlorosis, reddening, size reduction, deformation and plant dwarfing (Figure 2F–I), and seed production is diminished. During 1994–1999 in southwest WA, surveys of experimental plots of pulse cultivars found CMV infecting symptomatic plants of chickpea, field pea, faba bean and lentil (Figure 7A–D), and the minor pulses narbon bean, grass pea and dwarf chickling [105,161]. Also, during 1994–1999 in the same region, larger-scale surveys of commercial chickpea, field pea, faba bean and lentil crops found the incidence of CMV-infected crops within any one year was up to 23% in lentil, 17% in chickpea, 6% in field pea and 7% in faba bean. However, within-crop CMV incidence was generally low, being greatest in lentil at 36%. Seed-borne CMV infection at levels of up to 2% and 1% of germinated seedlings was found in chickpea and lentil seed lots, respectively. CMV seed transmission from harvested seeds to seedlings of both pulses was found in seed samples. In lentil this occurred not only in seeds from commercial crops but also in the seeds of advanced selections nearing release as new cultivars. As with seed-infected narrow-leafed lupin plants found occurring naturally in the field (Figure 3C–K), seed-infected plants of lentils were severely dwarfed and lacked the normal-looking lowermost leaves usually found in current-season-infected plants (Figure 7E). In 1998 and 1999, CMV was detected in 1/30 field pea, 1/11 faba bean and 3/50 chickpea commercial seed stocks [105,161].

In 2006, surveying crops of field pea in VIC and southern NSW, and faba bean in NSW, detected CMV in (i) 0/21 and 2/10 crops of field pea from NSW and VIC, respectively, at an incidence of 1–2% in the two infected crops found; and (ii) 0/3 faba bean crops from NSW [162]. In 2012, 12 chickpea crops growing in the Liverpool Plains were surveyed by collecting and testing 240 symptomatic and 159 asymptomatic samples [163]. CMV was detected in eight (=3%) of symptomatic leaf samples from 6/12 crops, but none of the 159 asymptomatic samples. In addition, when an additional 469 symptomatic and 1731 asymptomatic leaf samples from 2200 random samples collected from these 12 crops were tested, CMV was detected in only 6 (=1.3%) of the symptomatic samples and 1 (=0.1%) of the asymptomatic samples [163]. No CMV was detected in volunteer lucerne (=alfalfa) plants or adjacent lucerne pastures and faba bean crops, suggesting these were unlikely to be reservoirs for its spread to chickpea [163]. In 2013, when samples from chickpea plants showing viral symptoms were sampled at different locations in the NSW Liverpool Plains region, CMV was found at 18/18 locations at incidences of 2–8% [164]. During 2000–2007, annual surveys of lentil, faba bean and chickpea crops in VIC and SA also detected CMV but were only published in brief conference abstract format, so details of the extent of crop infection are not available [107,165,166,167,168]. In 2021, CMV was isolated from a single faba bean sample from VIC and complete sequences of all three components of its tripartite genome (RNA1, RNA2 and RNA3) were obtained [169].

#### 3.2.2. Seed Yield Losses and Patterns of Spread

Yield loss data for CMV infection in lentil cv. Matilda were obtained from a WA field experiment in 1998 in which the virus was spread within plots from introduced infection foci sown with a CMV-infected narrow-leafed lupin seed stock to healthy plants by naturally occurring aphids [170]. Infected plants were labelled individually using coloured ribbons to denote when CMV symptoms (leaf chlorosis and distortion followed by plant dwarfing) first appeared. Paired healthy and infected plant comparisons revealed individual seed yield losses of 80–90% and a reduction of 17–25% in individual seed weight [170]. Seed yield loss data for CMV infection of chickpea were provided by two replicated field experiments in which infection foci were introduced to simulate seed-infected plants growing within plots; a non-host canola buffer surrounded each plot [97]. In exp 1 in 1999, chickpea infector plants were transplanted in a single file along the middle of each rectangular plot (2.8 × 10 m) of chickpea cv. Sona. They were spaced the same distance apart within each treatment to simulate 0.3%, 0.5%, 1% and 2% CMV seed infection (Figure 7F). A plot without any infector plants simulated 0% initial seed-borne infection (healthy control) (Figure 7G). Within each plot, naturally occurring aphid vector migrants spread CMV from infection foci to healthy plants (there was no aphid colonisation). Plots with 1–2% initial simulated CMV incidences reached 61–74% infection at the final assessment (Figure 7H), and their seed yields were diminished by 44–45%. There was no significant seed yield decrease in plots with 0.3–0.5% simulated initial CMV incidences in which its spread was slower, eventually reaching final incidences of 47–63%. In exp 2 in 1997, four square plots (20 × 20 m) of chickpea cv. Tyson were sown. In two plots, five infection foci were introduced by sowing the seed of a CMV-infected narrow-leafed lupin seed stock (c. 50 seeds/seed stock) close to each plot corner and in its centre. The other two plots lacked CMV infection foci (control plots). Different coloured tape was used to mark individual plants that developed symptoms at different stages of growth (Figure 7I). When the seed yields of individual infected plants were compared with those individual plants without any symptoms, early and late CMV infection diminished seed yields by 78–80% and 65–67%, respectively. Individual seed weight was diminished by 20–25%. Seed size remained unchanged, so the yield difference between plants infected at different growth stages was all due to a reduction in seed number. However, CMV infection also caused seed malformation and discolouration, so it impaired seed quality. Information on temporal CMV spread patterns in chickpea stands was also obtained from these field experiments. Pathogen progress curves were sigmoid, and the rate of virus spread depended on the size of the initial infection focus, with little CMV spread in plots lacking them. When the Spatial Analysis by Distance IndicEs (SADIE) program [148,149] was used to analyse the final individual plant data for CMV infection in exp 2, as with CMV spread in lupin (Section 3.1.6 above), the contour map obtained revealed a marked contrast between areas closest to each introduced infection focus source where patch clusters predominated, and areas furthest from the source where gap clusters predominated (Figure 7J). This reflected comprehensive localized CMV spread around introduced infection foci. By contrast, where introduced infection foci were absent, the limited CMV spread that occurred was diffuse, producing mostly tiny patch clusters intermingling with reasonable-sized gap clusters. This reflected CMV spread by aphid vectors over the non-host buffer from neighboring plots with infection foci [97].

#### 3.2.3. Host Resistance

In an SA study published in 1992, the responses of single cultivars of lentil, faba bean, common bean and cowpea, and of 1–6 cultivars each of three clovers and six annual medic species, to inoculation with 16 CMV isolates from diverse host species were recorded [122]. Apart from an annual medic species that remained uninfected (*M. scutellata*), all species inoculated became infected by at least four isolates. The resulting phenotypes varied from symptomless systemic infection in common bean and infection restricted to necrotic local lesions in inoculated leaves of faba bean to severe systemic symptoms with two isolates in cowpea and within most isolates in all pasture species apart from white clover (*T. repens*). In WA, from 1994 to 1998, seven field screening experiments examined the susceptibilities and sensitivities of different pulse species to CMV infection [106]. The majority of the 39 lentil and 24 chickpea genotypes evaluated were susceptible or highly susceptible, but one chickpea (cv. Amethyst) and eight lentil genotypes had moderate CMV resistance, ILL7163 being the most resistant. All chickpea genotypes were ranked sensitive or highly sensitive, but the sensitivity rankings in lentil genotypes ranged from low to high. By contrast, the field pea and faba bean genotypes were all resistant or highly resistant, although their sensitivity rankings differed, with faba bean genotypes all being highly sensitive, whereas the field pea genotypes were all tolerant. Only two species of the other pulses evaluated were susceptible, the rankings for their different genotypes being susceptible to moderately resistant (narbon bean) and susceptible (bitter vetch). None of the eight other species became infected (common vetch, grass pea, dwarf chickling, *Lathyrus clymenum*, *L. ochrus*, *L. tingitanus*, purple vetch and *V. monantha*). Bitter vetch was very sensitive, but narbon bean had intermediate sensitivity. Seed-borne CMV transmission to seedlings was detected in narbon beans (0.1–0.8%), lentils (0.3%) and chickpeas (0.2–0.3%). When 16 pasture or forage legume species (1 genotype each) were evaluated in two field experiments, their susceptibility rankings ranged from highly susceptible to highly resistant, with only one remaining uninfected (*Ornithopus sativus*). The sensitivities of the 15 susceptible species varied from low to very high. Also, seed transmission at rates of 0.04–5% occurred in eight of them [106]. These findings suggested that a wide spectrum of pasture species may act as CMV reservoirs for its spread to pulse crops. A VIC study in 2001–2002 added information concerning potential alternative host reservoirs for CMV spread to pulse crops other than lupins by identifying the weed species carrot weed (*Bifora testiculata*), prickly lettuce (*Lactuca serriola*), sowthistle (*Sonchus oleraceous*) and eastern rocket (*Sisymbrium orientale*) and the pasture species burr medic as hosts [171]. In earlier studies in SA, WA and VIC, CMV was found to infect and be seed-borne in burr medic [18,120,124,127,172].

As with narrow-leafed lupin (Section 3.1.3 above), a cornerstone of effective management of CMV infection in the other cool-season pulse crops grown in Australia in which this virus is seed-borne is likely to be sowing healthy seed stocks and avoiding the release of any new cultivars with seed-borne infection. However, no Australian studies investigating the effects of phytosanitary or cultural control measures for CMV management have been undertaken as yet with these pulses. As with narrow-leafed lupin (Section 3.1.4 above), chemical control of aphid vectors with insecticides is unlikely to act quickly enough to prevent probing and virus inoculation of the treated plants [50]. In relation to possible control through host resistance, in studies under Australian conditions, chickpea and lentil were more CMV susceptible than field pea and faba bean, and the virus caused major seed yield losses in both of them. No CMV resistance genes are reported for cool-season pulses overseas [50]. However, although no CMV resistance was found in chickpea, Australian studies identified potentially useful CMV resistance to be present in some lentil genotypes (see above in this section).

#### 3.2.4. Further Research

With the exception of narrow-leafed lupin, CMV infects lentil most often among the other major cool-season pulses, increases to higher incidences in this pulse, and not only causes major yield losses but also is seed-borne. Therefore, further research is warranted on CMV in lentil. The main future focus should be on (i) conducting regular annual surveillance of CMV occurrence in lentil seed stocks and crops to establish its current prevalence; (ii) devising procedures to produce healthy commercial lentil seed stocks resembling the measures previously employed to minimise narrow-leafed lupin seed stock infection [44,112,120] (Section 3.1.1 above); (iii) establishing safe CMV infection threshold levels appropriate for commercial lentil seed stocks destined for sowing in different Australian grainbelt regions [61] (Section 3.1.3 above); (iv) utilising the most effective moderately CMV-resistant genotype identified (ILL7163) to breed new CMV-resistant lentil cultivars for Australian conditions; (v) searching for lentil genotypes with greater CMV virus resistance to use in Australian lentil breeding; (vi) identify cultural control measures likely to be effective at reducing CMV spread in lentil crops by maximising shading out seed-infected source plants or suppressing landings of alate aphid vectors, e.g., finding optimal row spacing, extents of stubble retention and seeding rate levels [44] (Section 3.1.3 above); and (vi) developing IDM packages effective in suppressing CMV spread in commercial lentil crops growing in different grainbelt regions.

## 4. Alfalfa Mosaic Virus

**Figure 8:**
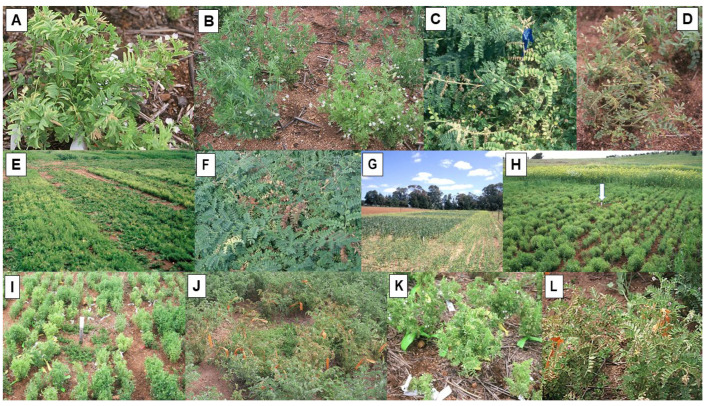
Australian field studies with alfalfa mosaic virus (AMV) infecting the cool-season pulses lentil, faba bean and chickpea. (**A**), Plant of lentil cv. Matilda showing foliage symptoms of leaflet chlorosis, mild mosaic, downcurling and size reduction caused by infection with AMV (Avondale 1998). (**B**), Plant of lentil cv. Matilda showing foliage symptoms of leaflet chlorosis, downcurling and size reduction, along with plant dwarfing caused by infection with AMV (front right), other plants all healthy (Avondale 1998). (**C**), Plant of Kabuli chickpea showing foliage symptoms of leaflet chlorosis and shoot necrosis caused by infection with AMV (bottom right), other plants healthy (South Perth 1996). (**D**), Plant of Desi chickpea showing foliage symptoms of leaflet necrosis and reddening caused by infection with AMV (Avondale 1999). (**E**), Experimental plots of lentil showing foliage symptoms of chlorosis and plant dwarfing caused by infection with AMV (Cunderdin 1997). (**F**), Experimental plot of desi chickpea showing foliage necrosis and reddening (central region) caused by infection with AMV (South Perth 1996). (**G**), Example of field experimental layout used in 1998–1999 for field experiments designed to quantify seed yield losses caused by AMV in lentil, chickpea and faba bean; square (20 × 20 m) plots were surrounded by 5 m wide non-host canola buffers (Faba bean cv. Fiord experiment, Medina 1998). (**H**), Portion of experimental field plot with surrounding canola buffer behind it from yield loss experiment with lentil cv. Matilda; stake indicated by white arrow marks position of introduced AMV infection focus and dwarfed plants with chlorotic foliage are AMV-infected (Avondale 1998). (**I**), Central portion of experimental plot of lentil cv. Matilda showing AMV infector plant focus consisting of infected burr medic transplants surrounding central white stake; AMV-infected lentil plants are those with chlorotic foliage and plant dwarfing and different coloured ribbon tags indicate when symptoms first appeared (Avondale 1998). (**J**), Central portion of experimental plot of desi chickpea cv. Tyson showing central infector plant focus consisting of AMV-infected medic transplants surrounded by AMV-infected chickpea plants having shoots with leaflet necrosis and reddening (orange-coloured ribbon tags), plants surrounding them had not yet developed symptoms (Avondale 1999). (**K**), Plants of lentil cv. Matilda with foliage symptoms of leaflet chlorosis, downcurling and reduced size, and plant dwarfing caused by AMV infection; infected plants dwarfed to different extents based on whether symptoms first appeared earlier (white ribbons) or later (green ribbons) (Avondale 1998). (**L**), Plants of chickpea cv. Tyson with foliage symptoms of leaflet necrosis and reddening and apical shoot necrosis caused by AMV infection; orange tags record when symptoms first appeared (Avondale 1999).

The first record of AMV in Australia was in 1945 [18,173], but it remained uncommon until the late 1970s when AMV-contaminated seed stocks of aphid-resistant lucerne cultivars were imported on a large scale from the USA. The aim of these lucerne importations was to address the recent incursion and subsequent spread of three lucerne aphid species, *A. kondoi*, *A. pisum* and *T. trifolii* [174]. These three aphid species and several others transmit AMV non-persistently [18,175]. In WA, where little lucerne is grown, AMV remained uncommon until the 1980s, when it became widely dispersed due to the widespread sowing of AMV-contaminated annual medic seed stocks in pastures [176]. By 1988, AMV was present in QLD, NSW, VIC, SA, WA and TAS and was reported to infect the cool-season pulses chickpea, field pea, lentil, narbon bean and narrow-leafed lupin [18]. The symptoms AMV causes in infected pulse plants vary widely in type and severity. They commonly consist of leaflet mosaic and deformation and plant dwarfing as in lentil or apical foliage necrosis, sometimes followed by plant death as in chickpea (Figure 8A–D) (Table 1).

### 4.1. Pulses Other Than Lupins

#### 4.1.1. Occurrence in Plots, Crops and Seed Stocks

In 1994–1999, in southwest WA, surveys of experimental plots found AMV infecting symptomatic plants of the major pulses chickpea, field pea and lentil (Figure 8E,F), and the minor pulses narbon bean, grass pea and fenugreek [106,161]. During the same period and in the same region, larger-scale surveys of commercial chickpea, field pea, faba bean and lentil crops found the incidence of AMV-infected crops within any one year was up to 23% in lentil, 5% in chickpea, 15% in field pea, and 7% in faba bean, but the within-crop incidence was always low, being greatest in lentil at 9% [106,161]. In 1996, AMV was also detected infecting symptomatic chickpea plants growing in experimental plots in tropical northwest WA [177]. In seeds of chickpea and lentil from southwest WA, AMV was detected at incidences of up to 2% for chickpea and 5% for lentil. These samples were from commercial crops or advanced selections nearing release as new cultivars. Also, seed-borne AMV and CMV were found to occur together in some lentil and chickpea seed samples [161]. A lentil seed sample received from VIC had 0.2% AMV infection of grown-out seedlings [105].

Several surveys found AMV infection occurring at low incidences in chickpea and faba bean crops growing in the Liverpool Plains, NSW. For example, when faba bean crops were surveyed in 2001, AMV was detected in 7/10 crops, but its within-crop incidence never exceeded 2% [178]. In 2006, a survey of field pea crops in southern NSW and VIC, and of faba bean crops in NSW, detected AMV in: (i) 1/21 and 0/10 field pea crops from NSW and VIC, respectively, occurring at an incidence of 1% in the single AMV-infected crop found; and (ii) 2/3 faba bean crops from NSW, both at an incidence of 1% [162]. In 2012, 12 chickpea crops growing in the Liverpool Plains were surveyed by collecting and testing 240 symptomatic and 159 asymptomatic samples [163]. AMV was detected in 19 (=13%) symptomatic samples from 7/12 crops but in none of 159 asymptomatic samples. However, when an additional 469 symptomatic and 1731 asymptomatic samples from among 2200 random samples collected from these 12 crops were tested, AMV was detected in 28 (=6%) of the symptomatic samples and 39 (=2%) of the asymptomatic samples [163]. Moreover, AMV was detected in volunteer lucerne plants and in adjacent lucerne pastures or faba bean crops, suggesting these were likely virus reservoirs for its spread to chickpea [163]. In 2013, when samples from chickpea plants showing viral symptoms were sampled at different locations in the Liverpool Plains region of NSW, AMV was detected at 18/24 locations at incidences of 5–88% [164]. In 2000–2007, annual surveys of lentil, faba bean and chickpea crops in VIC and SA sometimes also detected AMV. These findings were published only in brief conference abstract format, so details of the extent of crop infection are unavailable. However, ‘high infection incidences’ were present in lentil, including in lentil seed stocks [107,165,166,167,168]. In 2019, AMV was detected by metagenomic analysis in a single field pea sample from NSW, and a nearly complete AMV genome was obtained [179].

#### 4.1.2. Seed Yield Losses

In WA in 1998–1999, field experiments with AMV provided yield loss data from replicated plots of chickpea, faba bean and lentil (Figure 8G,H) in which naturally occurring aphids spread virus infection from introduced infection foci to healthy plants [170]. The infector plants introduced consisted of transplants grown from infected burr medic seedlings derived from an AMV-infected seed stock. When typical AMV symptoms first appeared in individual infected chickpea, faba bean or lentil plants, they were labelled using coloured ribbons to denote when their symptoms first appeared (Figure 8I–L). Yield loss parameters were established using paired healthy and infected plant comparisons. The foliage symptoms that developed in early-infected faba bean plants (chlorotic mottle, marginal curling, size reduction and necrotic spots in leaves and plant dwarfing) were followed by recovery in later growth (symptomless infection). In consequence, no statistically significant seed yield losses from early AMV infection of faba bean were recorded. By contrast, early-infected chickpea plants were killed, so the seed yield losses recorded for them were 100%. The seed yield losses recorded for late infection in faba bean and chickpea were 45% and 98%, respectively. When lentil plants became AMV-infected at different growth stages, their yield losses were 81–87%. Individual seed weight was diminished by 10–21% in lentil and 90% in chickpea, but no data were collected for this parameter with faba bean. A low rate of AMV seed transmission (0.04%) to seedlings was detected in faba bean [170].

#### 4.1.3. Host Resistance

**Figure 9:**
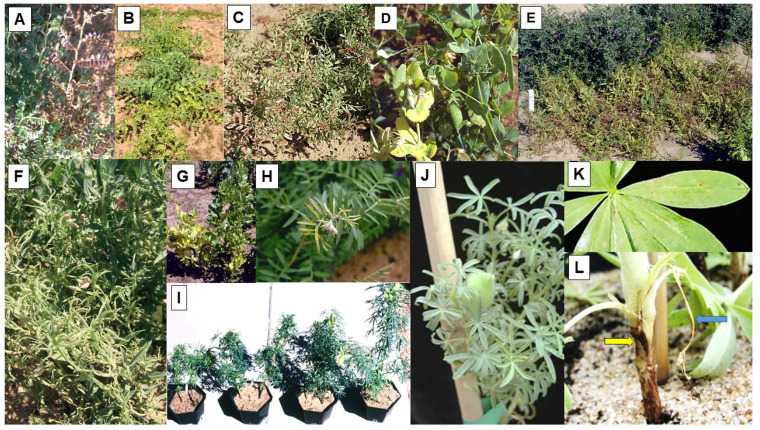
Screening for susceptibility and sensitivity to alfalfa mosaic virus (AMV) in major and minor cool-season pulse species at Medina, southwest Australia in 1998 (**A**–**H**), and studies on host resistance to AMV in lupin species at South Perth in 1996 or 2006 (**I**–**L**). (**A**), Single shoots of Desi chickpea showing foliage symptoms of leaflet reddening and necrosis caused by AMV infection (right) or without symptoms (left). (**B**), Row of Kabuli chickpea plants showing chlorotic foliage symptoms caused by AMV infection (front and rear) or without symptoms (central). (**C**), Portion of lentil row consisting of plants with foliage symptoms of leaflet chlorosis, reddening, downcurling and necrosis caused by AMV infection. (**D**), Portion of field pea row with foliage symptoms of yellow leaflet chlorosis and necrosis caused by AMV infection (left) or lacking symptoms (right). (**E**), Row of dwarf chickling plants showing foliage symptoms of leaflet chlorosis, curling and size reduction in upper leaves, necrosis in lower leaves and plant dwarfing caused by AMV infection; annual pasture legume row behind lacked symptoms. (**F**), Portion of grass pea row showing foliage symptoms of leaf chlorosis, deformation and size reduction and plant dwarfing caused by AMV infection (front), or without symptoms (back). (**G**), Two narbon bean plants showing foliage symptoms of yellow leaflet chlorosis, deformation and size reduction, and plant dwarfing caused by AMV infection (left and bottom right corner) and two tall plants without symptoms (central and right). (**H**), Shoot of common vetch with apical shoot necrosis caused by AMV infection. (**I**), Plants of narrow-leafed lupin cv. Gungurru (2/pot) sap inoculated with AMV and/or cucumber mosaic virus (CMV); plants inoculated with both viruses show severe dwarfing (left), plants inoculated with CMV alone show moderate dwarfing (centre left), plants inoculated with AMV alone show mild dwarfing (centre right), uninoculated healthy control plants without symptoms (right) (1996). (**J**), Shoot of narrow-leafed lupin cv. Tanjil sap inoculated with AMV showing foliage symptoms of leaflet chlorosis, faint mosaic, downcurling and size reduction (2006). (**K**), Leaf of pearl lupin plant sap inoculated with AMV showing localized hypersensitive phenotype consisting of necrotic local lesions (brown spots) (2006). (**L**), *Lupinus digitatus* plant graft inoculated (side graft) with an AMV-infected scion (now dead, blue arrow); localized hypersensitive phenotype consisting localized stem necrosis just below the graft union (yellow arrow) (2006). Images (**D**,**G**,**H**) modified from Latham and Jones [180], (**J**–**L**) modified from Jones [51].

In WA in 1998, a field screening experiment examined the susceptibilities and sensitivities of pulses other than lupins to AMV infection [180]. Using a randomised block replicated design, 1.5 m long single-row plots were sown, and AMV-infected infector plants or healthy plants of burr medic were transplanted at each end of every test row. Naturally occurring aphid vectors spread the virus from infector plants to healthy plants within the test rows. Every test row was inspected on a regular basis, and both symptom expression and relative susceptibility and sensitivity rankings were allocated to each genotype. Examples of the diverse symptom types recorded in major and minor cool-season pulses are shown in Figure 9A–H. The 23 different chickpea genotypes included were all highly susceptible. Among the 19 lentil genotypes, the rankings were nine as highly susceptible, eight as susceptible and one each as moderately resistant (cv. Digger) and resistant (ILL5480). Three faba bean and five field pea genotypes were susceptible, moderately resistant or resistant, but one faba bean genotype (cv. Ascot) remained uninfected. Genotypes of narbon bean (5), grass pea (5), dwarf chickling (5), common vetch (1), *Lathyrus ochrus* (2) and purple vetch (*Vicia benghalensis*) (1) were highly susceptible, susceptible, or moderately resistant. Sensitivity rankings ranged from high in some genotypes of all species tested apart from purple vetch to low in *L. ochrus*. In glasshouse inoculations, faba bean cv. Ascot became systemically infected. AMV was seed-borne in common vetch (0.7%), narbon bean (0.1%), grass pea (0.9–4%), dwarf chickling (2%), and purple vetch (0.9%). Twenty genotypes (19 species) of pasture and forage legumes were included in these studies. Their susceptibility rankings ranged from highly susceptible to resistant, with only one species remaining uninfected, and their sensitivities varied from very high to low. In addition, AMV was seed-borne at rates of 0.05–7% in 15 of them [180]. This suggested that a wide spectrum of pasture species may act as reservoirs for AMV spread to pulse crops.

#### 4.1.4. Alternative Hosts

Earlier records of AMV infecting weed and pasture host species from QLD, NSW, VIC, SA, TAS and/or WA are listed by Buchen-Osmond et al. [18]. These include pasture species, such as white clover and red clover (*T. pratense*), and weeds such as stagger weed, cape gooseberry (*Physalis peruviana*), fat hen (*Chenopodium album*) and siratro (*Macroptilium atropurpureum*). In addition, a WA study in 1987–1993 found AMV naturally infecting many pasture legume species and 10 weed species, the most commonly infected weeds being spreading stonecrop, King Island melilot and flatweed [181]. Seed transmission of AMV to seedlings was found in several pasture legume species, including annual medics, serradella, subterranean clover and lucerne, and the weeds King Island melilot, flatweed, spreading stonecrop and stagger weed [127,176,181,182]. AMV’s ability to survive in dormant seeds over the hot, dry summer period and infect their seedling progenies in the following autumn provides an important means for it to persist between successive years in pulse-growing regions of Australia with Mediterranean-type climates, such as southwest WA [61,62]. A VIC study in 2001–2002 also contributed to the knowledge of alternative host reservoirs for AMV spread to pulse crops by identifying volunteer lucerne and the weed species sowthistle and blackberry nightshade (*Solanum nigrum*) as hosts likely to act in this way [171].

#### 4.1.5. Further Research

Since AMV reaches high infection incidences in lentil crops, causes considerable yield losses in this pulse, and is readily transmitted through its seed, further studies are required with AMV in lentil. The main focus of these studies should involve: (i) developing methodologies for healthy commercial lentil seed stock production like those used to minimize CMV infection in narrow-leafed lupin seed stocks [44,112,120] (Section 3.1.1 above); (ii) establishing safe threshold levels for AMV infection appropriate for commercial lentil seed stocks destined for sowing in different Australian grainbelt regions [61] (Section 3.1.3 above); (iii) utilizing the AMV-resistant lentil genotype identified (ILL5480) in breeding AMV-resistant lentil cultivars for Australia; (iv) searching for further sources of AMV-resistance suitable for use in Australian lentil breeding programs; (v) field experimentation to establish which cultural control measures are likely to be effective, e.g., establishing the optimal seeding rates, row spacing and stubble retention levels to shade out seed-infected plants or suppress aphid vector landings [44] (Section 3.1.3 above); and (vi) devising comprehensive IDM packages, which include appropriate phytosanitary, cultural and host resistance control measures, that operate in different ways, thereby ensuring that the control of AMV spread is effective in commercial lentil crops growing in different grainbelt regions.

### 4.2. Lupins

#### 4.2.1. Research Findings

AMV has been reported to infect white lupin naturally in Europe [49]. Within Australia, however, naturally occurring AMV infection has only been reported in narrow-leafed lupin in VIC and SA [18,49,183]. In WA glasshouse studies, when nine lupin species were inoculated with infective sap or by graft inoculation, narrow-leafed lupin, yellow lupin and *L. hispanicus* developed susceptible phenotypes resulting in systemic infection [184]. In narrow-leafed lupin, the predominant leaf symptoms were mild mosaic, downcurling and reduced size, and the affected plants were stunted (Figure 9I,J). These symptoms were similar to those caused by CMV (Figure 2A,B and Figure 3H,K) but milder. However, AMV and CMV co-infection caused symptoms that were more severe than those that occurred when each of them was present on their own (Figure 9I). In yellow lupin, systemic infection was asymptomatic, whereas *L. hispanicus* plants developed occasional leaf necrotic line patterns or mild plant dwarfing. Pearl lupin, *L. atlanticus* and *L. digitatus* developed LHR phenotypes following sap (Figure 9K) or graft inoculation (Figure 9L). Although plants of white lupin and *L. pilosus* were not infected by sap inoculation, some plants developed localized (white lupin) or systemic hypersensitive (*L. pilosus*) phenotypes following graft inoculation. Sandplain lupin never became infected. AMV seed transmission at 0.8% was found in a narrow-leafed lupin seed sample from SA [184].

Close proximity to AMV-infected annual medic and lucerne pastures [183], sowing AMV-contaminated seed stocks of these pasture legumes, or both, likely play critical roles as primary inoculum sources for AMV spread to narrow-leafed lupin crops in SA and VIC. Although AMV has not been found infecting lupin crops in WA, a thorough survey is required to establish whether this occurs. Its presence seems likely, especially in lupin crops adjacent to annual medic or lucerne pastures, as these are often AMV-infected due to widespread contamination with this virus among sown seed stocks [45,46,177,182].

#### 4.2.2. Further Research

Since the symptoms AMV causes in narrow-leafed lupin foliage resemble those caused by CMV in this crop, it is likely they are being attributed to CMV, resulting in an underestimation of its importance in this pulse species. Establishing the extent and importance of AMV infection in commercial crops of narrow-leafed lupin, therefore, warrants serious attention, especially in SA, where sources of AMV infection in lucerne and annual medic pastures often occur in close proximity to lupin crops. The extent of AMV infection in narrow-leafed lupin seed stocks also needs to be determined not only in SA but also in WA where information is entirely lacking on this issue. Furthermore, lupin breeding programs may unknowingly be releasing AMV-infected seed stocks of new narrow-leafed lupin cultivars, so the extent to which this is occurring needs to be investigated.

## 5. Less Important Pulse Viruses

BBWV was first reported to cause wilting in faba bean in 1947 in VIC [185]. It was subsequently found in NSW, QLD and TAS, and in other continents. Its natural Australian hosts also include the pulses narrow-leafed lupin, white lupin, field pea, chickpea, common bean and cowpea and species in several other families (Table 1) [18,186,187]. In faba bean, it causes initial foliage symptoms of vein clearing, followed by plant wilting and sometimes plant death, but infected plants sometimes recover, then developing leaf distortion and mottle. In field pea, BBWV causes severe wilting, apical necrosis and axillary shoots develop leaf mosaic and deformation and stunted growth. Its ‘pea streak’ strain causes somewhat different symptoms in pea, including necrotic stem streaking. BBWV is transmitted non-persistently by aphid vectors [18,186] and is seed-borne at low rates (0.4–0.6%) to seedlings of faba bean [188]. Based on nucleotide sequence differences, later studies separated BBWV into two viruses, BBWV-1 and BBWV-2 [25,187], although they were indistinguishable by host range, host symptoms and transmission by aphids. BBWV-2 occurs more commonly in Australia, Asia and North America, while BBWV-1 is common in Europe [25,187]. Neither virus causes diseases considered economically significant in Australian pulse crops. BBSV and BBTMV were first reported in Australia, infecting faba bean in the early 1970s. They were found in the ACT in seeds bought in Canberra and in SA in seeds imported from the U.K. [18,189,190]. In faba bean, they both cause leaf chlorotic mottling and deformation, sometimes accompanied by shoot necrosis, and seeds are deformed, developing necrotic seed staining and deformation. Both BBTMV and BBSV are readily seed-borne in this host (seed transmission up to 10% for BBSV and 15% for BBTMV) and have beetle vectors. Both infect field pea and common bean experimentally [18,191,192]. In Australia, no other hosts or new detections in faba bean have been reported for either virus since the 1970s. Therefore, both are considered no longer present in Australian pulse crops, so BBTMV and BBSV are now treated as biosecurity threats. In 1966, PMoV was detected, causing a chlorotic foliar mottle in peanut and pea plants in QLD [193]. It was seed-borne in peanut, infecting up to 7% of seedlings, and non-persistently transmitted by aphids. PMoV was later found infecting the pulses common, navy, adzuki and lima bean and soybean in QLD, but the only cool-season pulse infected was pea [18,194]. In 2016, in QLD, leaf mottle and pod deformation symptoms in plants of common bean and soybean were shown to be caused by CpMMV [65,66]. In other continents, CpMMV is also found naturally infecting cowpea, mung bean and pea [195]. CPMMV is seed-borne in soybean, cowpea and common bean, and, unusually for a carlavirus, it is whitefly transmitted instead of by aphids [195]. Neither PMoV nor CpMMV currently causes economically significant diseases in Australian cool-season pulse crops.

## 6. Conclusions

Here, we provide the first volume of a three-part series of historical review articles that describe Australian research performed on seed-borne virus diseases of cool-season pulse crops during the period 1950–2023. A comprehensive account is provided of past investigations involving the occurrence, epidemiology and management of two (AMV and CMV) of the four most damaging seed-borne viruses in the cool-season pulse crops chickpea, faba bean, field pea, lentil and lupin. Brief descriptions of the limited past Australian studies on infection of minor cool-season pulse crops with AMV and CMV and of cool-season pulse crops with the minor viruses BBSV, BBTMV, BBWV, CMMV and PMoV are also included. Since more in-depth Australian studies were performed with the lupin/CMV pathosystem than with any of the other host–virus combinations, it is covered in the greatest detail. This coverage includes (i) virus incidence surveys in commercial crops and seed stocks, and in both breeding and other types of experimental plots; (ii) alternative host infection reservoir and aphid vector studies; (iii) investigations directed at managing CMV infection during lupin breeding activities; (vi) the extensive series of field and glasshouse experiments that provided yield loss information and enabled the development of effective phytosanitary, cultural and host resistance management strategies; (vi) field experiments on chemical control of aphid vectors; (vii) studies of the temporal and spatial dynamics of CMV spread, and factors driving CMV epidemic development in different grainbelt regions; (viii) the formulation of integrated disease management strategies suited to commercial crops growing in different grainbelt regions or to high-value seed crops; and (ix) the development of a forecasting model and DSS for large-scale use by the lupin industry.

Australian research undertaken on CMV infection in cool-season pulses other than lupin and on AMV infection in all cool-season pulses has been less comprehensive. It includes virus incidence surveys in crops, experimental plots and seed stocks, alternative infection reservoir and aphid vector studies, obtaining yield loss data from field experiments, evaluation of genotypes for their vulnerability (relative susceptibility and sensitivity) and resistance to infection, and limited studies (only with CMV in chickpea) on the temporal and spatial dynamics of virus spread. However, in other areas, it lacks any of the in-depth studies performed with the lupin/CMV pathosystem, including field and glasshouse experimentation directed at developing effective phytosanitary and cultural control strategies, formulation of effective integrated disease management strategies, and development of forecasting models and DSS’s.

Section 2.4 provides general research recommendations for future Australian cool-season pulse virus research. Future research priorities for the lupin/CMV pathosystem, CMV infection of other cool-season pulses and AMV infection in cool-season pulses are provided elsewhere within the text (Section 3.1.5, Section 3.1.7, Section 3.1.8, Section 3.2.4, Section 4.1.5 and Section 4.2.2). In summary, with the lupin/CMV pathosystem, there is a need to focus not only on ensuring the effective incorporation of already known CMV host resistances into new lupin cultivars but also on identifying new resistance sources for use in future lupin breeding. Genetic modification for CMV resistance involving RNA silencing and genome editing is also needed, along with streamlining the breeding process using speed breeding and deploying molecular markers. Another research priority is updating the existing forecasting model and DSS for CMV in lupin to include automated daily weather data retrieval and adjusting it to accommodate earlier sowing dates, increased climate instability, local agronomic practices and climatic conditions in different Australian national grainbelt regions. In addition, a vigorous extension effort is required focused on reminding farmers and lupin breeders about the need for greater vigilance over getting their lupin seed stock samples tested for CMV and ensuring that farmers apply established threshold levels for percentage seed infection correctly at sowing time. With the lupin/AMV pathosystem, the extent of seed-borne infection in commercial lupin crops and seed stocks, and the importance of nearby lucerne and annual medic pastures as AMV sources for spread to lupin crops, both need to be established, as does whether lupin breeding programs are releasing AMV-infected seed stocks of new cultivars. The outcome of such studies would then determine whether further action is warranted. Among the other cool-season pulses, infection of lentil with AMV and CMV warrants the most attention. The requirement here is firstly to develop effective methodologies for healthy commercial seed stock production and identify seed stock infection threshold levels safe for sowing in different Australian grainbelt regions. Next, it is to use currently available host resistances to both viruses to breed new virus-resistant cultivars suited to Australian conditions and search for lentil genotypes with improved virus resistance for future use in lentil breeding. Then, it is to identify cultural control measures (i.e., optimal row spacing, extent of stubble retention and seeding rates) that reduce CMV spread by maximising the shading out of seed-infected source plants or suppressing landings of winged aphid vectors. Finally, it is to develop IDM and DSS packages that effectively suppress virus spread in commercial lentil crops growing in different grainbelt regions and in lentil breeding programs.

Detailed historical accounts of past Australian research on the diseases caused by BYMV and PSbMV in cool-season pulses will be presented in the second and third volumes of this series.

## Data Availability

Not applicable for reviews based entirely upon previously published information.

## References

[1] Thresh J.M. (1980). The origins and epidemiology of some important plant virus diseases. Appl. Biol..

[2] Thresh J.M. (1982). Cropping practices and virus spread. Annu. Rev. Phytopathol..

[3] Thresh J.M., Cooper I., Kühne T., Polishchuk V.P. (2006). Crop Viruses and Virus Diseases: A Global Perspective. Virus Diseases and Crop Biosecurity.

[4] Bos L. (1982). Crop losses caused by viruses. Crop Prot..

[5] Jones R.A.C., Naidu R. (2019). Global dimensions of plant virus diseases: Current status and future perspectives. Ann. Rev. Virol..

[6] Jones R.A.C. (2020). Disease pandemics and major epidemics arising from new encounters between indigenous viruses and introduced crops. Viruses.

[7] Jones R.A.C. (2021). Global plant virus disease pandemics and epidemics. Plants.

[8] Bos L. (1992). New plant virus problems in developing countries: A corollary of agricultural modernization. Adv. Virus Res..

[9] Thresh J.M. (2006). Control of tropical plant virus diseases. Adv. Virus Res..

[10] Jones R.A.C. (2006). Control of Plant Virus Diseases. Adv. Virus Res..

[11] Jones R.A.C. (2009). Plant virus emergence and evolution: Origins, new encounter scenarios, factors driving emergence, effects of changing world conditions, and prospects for control. Virus Res..

[12] Jones R.A.C. (2014). Plant virus ecology and epidemiology: Historical perspectives, recent progress and future prospects. Ann. Appl. Biol..

[13] Jones R.A.C. (2016). Future scenarios for plant virus pathogens as climate change progresses. Adv. Virus Res..

[14] Jones R.A.C. (2018). Plant and insect viruses in managed and natural environments: Novel and neglected transmission pathways. Adv. Virus Res..

[15] Jones R.A.C., Barbetti M.J. (2012). Influence of climate change on plant disease infections and epidemics caused by viruses and bacteria. CAB Rev..

[16] Hull R. (2014). Plant Virology.

[17] Trębicki P. (2020). Climate change and plant virus epidemiology. Virus Res..

[18] Buchen-Osmond C., Crabtree K., Gibbs A.J., McLean G.D. (1988). Viruses of Plants in Australia: Descriptions and Lists from the VIDE Database (No. 632.8 V821v).

[19] Gibbs A.J., Mackenzie A., Wei K.J., Gibbs M.J. (2008). The potyviruses of Australia. Arch. Virol..

[20] Rodoni B.C. (2009). The role of plant biosecurity in preventing and controlling emerging plant virus disease epidemics. Virus Res..

[21] Gerritsen R. (2010). Evidence for indigenous Australian agriculture. Australas. Sci..

[22] Davis R.I., Jones L.M., Pease B., Perkins S.L., Vala H.R., Kokoa P., Apa M., Dale C.J. (2021). Plant virus and virus-like disease threats to Australia’s north targeted by the northern Australia quarantine strategy. Plants.

[23] Whattam M., Dinsdale A., Elliott C.E. (2021). Evolution of plant virus diagnostics used in Australian Post Entry Quarantine. Plants.

[24] Jones R.A.C., Sharman M., Trebicki P., Maina S., Congdon B.S. (2021). Virus diseases of cereal and oilseed crops in Australia: Current position and future challenges. Viruses.

[25] Maina S., Jones R.A.C. (2023). Enhancing biosecurity against virus disease threats to Australian grain crops: Current situation and future prospects. Front. Hortic..

[26] Boswell K.F., Gibbs A.J. (1983). Viruses of Legumes 1983; Descriptions and Keys from VIDE.

[27] Salam M.U., Davidson J.A., Thomas G.J., Ford R., Jones R.A.C., Lindbeck K.D., Macleod W.J., Kimber R.B., Galloway J., Mantri N. (2011). Advances in winter pulse pathology research in Australia. Australas. Plant Pathol..

[28] Food and Agriculture Organization (1994). Definition and Classification Commodities, 4. Pulses and Derived Products.

[29] Calles T. (2016). The International Year of Pulses: What Are They and Why Are They Important? Food and Agriculture Organization, United Nations. https://www.fao.org/3/bl797e/BL797E.pdf..

[30] Aykroyd W.R., Doughty J., Walker A.F. (1982). Legumes in human nutrition. United Nations Food Agric. Organ. Food Nutr. Pap..

[31] Singh N. (2017). Pulses: An overview. J. Food Sci. Technol..

[32] Herridge D.F., Peoples M.B., Boddey R.M. (2008). Global inputs of biological nitrogen fixation in agricultural systems. Plant Soil.

[33] Liu C., Plaza-Bonilla D., Coulter J.A., Kutcher H.R., Beckie H.J., Wang L., Floch J.B., Hamel C., Siddique K.H., Li L. (2022). Diversifying crop rotations enhances agroecosystem services and resilience. Adv. Agron..

[34] Siddique K.H.M., Sykes J. (1997). Pulse production in Australia past, present and future. Aust. J. Exp. Agric..

[35] Pulse Australia (2021). What Are Pulses?. https://www.pulseaus.com.au/using-pulses/what-are-pulses.

[36] Gladstones J.S. (1969). Lupins in Western Australia. 2. Cultivation methods. West. Aust. J. Agr. Fourth Ser..

[37] Siddique K.H.M., Loss S.P., Enneking D. (1996). Narbon bean (*Vicia narbonensis* L.): A promising grain legume for low rainfall areas of south-western Australia. Aust. J. Exp. Agric..

[38] Gladstones J.S., Atkins C.A., Hamblin J. (1998). Lupins as Crop Plants: Biology, Production and Utilization.

[39] French R.J., Sweetingham M.W., Shea G.G. (2001). A comparison of the adaptation of yellow lupin (*Lupinus luteus* L.) and narrow-leafed lupin (*L. angustifolius* L.) to acid sandplain soils in low rainfall agricultural areas of Western Australia. Aust. J. Agric. Res..

[40] Clements J.C., Buirchell B.J., Yang H., Smith P.M.C., Sweetingham M.W., Smith C.G., Singh R.J., Jauhar P.P.J. (2005). Lupin. Genetic Resources, Chromosome Engineering, and Crop Improvement. Grain Legumes.

[41] Clements J.C., Sweetingham M.S., Smith L., Francis G., Thomas G., Sipsas S., Palta J.A., Berger J.B. (2008). Crop Improvement in *Lupinus mutabilis* for Australian Agriculture—Progress and Prospects. Lupins for Health and Wealth, Proceedings of the 12th International Lupin Conference, Fremantle, Australia, 14–18 September 2008.

[42] Buirchell B.J., Palta J.A., Berger J.B. (2008). Narrow-Leafed Lupin Breeding in Australia—Where to from Here?. Lupins for Health and Wealth, Proceedings of the 12th International Lupin Conference, Fremantle, Australia, 14–18 September 2008.

[43] Jones R.A.C., Chackraborty S.L.K., Skipp R.A., Pederson G.A., Bray R., Latch G.C.M., Nutter F.W. (1996). Virus Diseases of Australian Pastures. Pasture and Forage Crop Pathology.

[44] Jones R.A.C. (2001). Developing integrated disease management strategies against non-persistently aphid-borne viruses: A model programme. Integr. Pest Manag. Rev..

[45] Jones R.A.C. (2012). Virus diseases of annual pasture legumes: Incidences, losses, epidemiology and management. Crop Pasture Sci..

[46] Jones R.A.C. (2013). Virus diseases of perennial pasture legumes: Incidences, losses, epidemiology and management. Crop Pasture Sci..

[47] Jones R.A.C. (2022). Alteration of plant species mixtures by virus infection: Managed pastures the forgotten dimension. Plant Pathol..

[48] Brunt A., Crabtree K., Gibbs A.J. (1990). Viruses of Tropical Plants: Descriptions and Lists from the VIDE Database.

[49] Jones R.A.C., McLean G.D. (1989). Virus diseases of lupins. Ann. Appl. Biol..

[50] Makkouk K.M., Kumari S.G., van Leur J.A.G., Jones R.A.C. (2014). Control of plant virus diseases in cool-season legume crops. Adv. Virus Res..

[51] Jones R.A.C. (2023). Host resistance to virus diseases provides a key enabler towards fast tracking gains in grain lupin breeding. Plants.

[52] Persley D.M., Cooke T., House S. (2010). Diseases of Vegetable Crops in Australia.

[53] Johnstone G.R., Barbetti M.J., Wheeler J.L., Pearson C.J., Roberts G.E. (1987). Impact of Fungal and Virus Diseases on Pasture. Temperate Pastures: Their Production, Use and Management.

[54] Panetta F.D., Ridsdell-Smith T.J., Barbetti M.J., Jones R.A.C., Delfosse E.S. (1993). The Ecology of Weeds, Invertebrate Pests and Diseases of Australian Sheep Pastures. Pests of Pastures: Weed, Invertebrate and Disease Pests of Australian Sheep Pastures.

[55] Barbetti M.J., Jones R.A.C., Riley I.T., Chakraborty S., Leath K.T.L., Skipp R.A., Pederson G.A., Bray R.A., Latch G.C.M., Nutter F.W. (1996). Problems and Progress in Assessing Direct and Indirect Yield Losses Caused by Pathogens in Pasture Species. Pasture and Forage Crop Pathology.

[56] Barbetti M.J., Pei You M.P., Jones R.A.C., de Brujin F. (2020). Medicago Truncatula and Other Annual *Medicago* spp.—Interactions with Root and Foliar Fungal, Oomycete, and Viral Pathogens. The Model Legume *Medicago truncatula*.

[57] Nichols P.G.H., Jones R.A.C., Ridsdill-Smith T.J., Barbetti M.J. (2014). Genetic improvement of subterranean clover (*Trifolium subterraneum* L.). 2. Breeding for disease and pest resistance. Crop Pasture Sci..

[58] Johnstone G.R., McLean G.D. (1987). Virus diseases of subterranean clover. Ann. Appl. Biol..

[59] Bos L., Hampton R.O., Makkouk K.M., Summerfield R.J. (1988). Viruses and Virus Diseases of Pea, Lentil, Faba Bean and Chickpea. World Crops: Cool Season Food Legumes.

[60] Edwardson J.R., Christie R.G. (1991). CRC Handbook of Viruses Infecting Legumes.

[61] Jones R.A.C. (2000). Determining threshold levels for seed-borne virus infection in seed stocks. Virus Res..

[62] Jones R.A.C. (2004). Using epidemiological information to develop effective integrated virus disease management strategies. Virus Res..

[63] Bos L., Mahy W.J., Van Regenmortel M.H.N. (2008). Legume Virology. Encyclopedia of Virology.

[64] Sastry K.S. (2013). Seed-Borne Plant Virus Diseases.

[65] Persley D.M., Steele V., Sharman M., Campbell P.R., Geering A.D., Gambley C. (2020). First report of a carlavirus infecting plants in the *Fabaceae* in Australia. New Dis. Rep..

[66] Gambley C., Nimmo P., Persley D., Steele V., Sharman M., Campbell P. (2022). Cowpea mild mottle virus, a sometimes problem for French bean crops. Australas. Plant Pathol..

[67] Henzell T. (2007). Australian Agriculture: Its History and Challenges.

[68] Brown A., De Costa C., Guo F. (2020). Our Food Future: Trends and Opportunities.

[69] Pratley J., Kirkegaard J. (2020). Australian Agriculture in 2020: From Conservation to Automation.

[70] Freund M., Henley B.J., Karoly D.J., Allen K.J., Baker P.J. (2017). Multi-century cool- and warm-season rainfall reconstructions for Australia’s major climatic regions. Clim. Past.

[71] Stephens D.J., Lyons T.J. (1998). Rainfall-yield relationships across the Australian wheatbelt. Aust. J. Agric. Res..

[72] Turner N.C., Wright G.C., Siddique K.H.M. (2001). Adaptation of grain legumes (pulses) to water-limited environments. Adv. Agron..

[73] Turner N.C., Asseng S. (2005). Productivity, sustainability, and rainfall-use efficiency in Australian rainfed Mediterranean agricultural systems. Aust. J. Agric. Res..

[74] Australian Export Grains Innovation Centre (2022). Australian Pulses. Australian Export Grains Innovation Centre, Sydney and Perth, Australia.

[75] Australian Export Grains Innovation Centre (2020). What Grows Where?. Australian Grain Production—A Snapshot. Australian Grain Note.

[76] Kimberley Development Commission (2020). Primary Industries: The Ord River Irrigation Area. https://kdc.wa.gov.au/economic-profile/primaryindustries/#:~:text=The%20Ord%20Valley%20produces%20a,stages%20over%20the%20last%20years.

[77] Northern Territory Government (2017). Agriculture, Forestry and Fishing. Northern Territory Economy. https://nteconomy.nt.gov.au/industry-analysis/agriculture,-foresty-and-fishing.

[78] Cooperative Research Centre for Northern Australia (2020). Northern Australian Broadacre Cropping Situational Analysis. https://www.crcna.com.au/resources/publications/northern-australian-broadacre-cropping-situationalanalysis.

[79] Grains Research and Development Corporation (2010). Australian Grains Focus 2010–2011.

[80] Jones R.A.C., Salam M.U., Maling T.J., Diggle A.J., Thackray D.J. (2010). Principles of predicting plant virus disease epidemics. Ann. Rev. Phytopathol..

[81] Jones R.A.C. (2014). Trends in plant virus epidemiology: Opportunities from new or improved technologies. Virus Res..

[82] Oerke E.C. (2020). Remote sensing of diseases. Annu. Rev. Phytopathol..

[83] Neupane K., Baysal-Gurel F. (2021). Automatic identification and monitoring of plant diseases using unmanned aerial vehicles: A review. Remote Sens..

[84] Rhodes M.W., Bennie J.J., Spalding A., Ffrench-Constant R.H., Maclean I.M. (2022). Recent advances in the remote sensing of insects. Biol. Rev..

[85] Wang Y.M., Ostendorf B., Gautam D., Habili N., Pagay V. (2022). Plant viral disease detection: From molecular diagnosis to optical sensing technology—A multidisciplinary review. Remote Sens..

[86] Croser J.S., Pazos-Navarro M., Bennett R.G., Tschirren S., Edwards K., Erskine W., Creasy R., Ribalta F.M. (2016). Time to flowering of temperate pulses in vivo and generation turnover in vivo–in vitro of narrow-leaf lupin accelerated by low red to far-red ratio and high intensity in the far-red region. Plant Cell Tissue Org. Cult..

[87] Zhao Y., Yang X., Zhou G., Zhang T. (2020). Engineering plant virus resistance: From RNA silencing to genome editing strategies. Plant Biotechnol. J..

[88] Taliansky M., Samarskaya V., Zavriev S.K., Fesenko I., Kalinina N.O., Love A.J. (2021). RNA-based technologies for engineering plant virus resistance. Plants.

[89] Tripathi L., Ntui V.O., Tripathi J.N., Abd-Elsalam K.A., Lim K.-T. (2021). RNA Interference and CRISPR/Cas9 Applications for Virus Resistance. CRISPR and RNAi Systems.

[90] Ding S.W. (2023). Transgene silencing, RNA interference, and the antiviral defense mechanism directed by small interfering RNAs. Phytopathology.

[91] Jones R.A.C. (2018). Progress in Understanding Pulse Disease Epidemiology and Management in Western Australia.

[92] Jones R.A.C., Proudlove W. (1991). Further studies on cucumber mosaic virus infection of narrow-leafed lupin (*Lupinus angustifolius):* Seed-borne infection, aphid transmission, spread and effects on grain yield. Ann. Appl. Biol..

[93] Bwye A.M., Jones R.A.C., Proudlove W. (1994). Effects of sowing seed with different levels of infection, plant density and the growth stage at which plants first develop symptoms on cucumber mosaic-virus infection of narrow-leafed lupins (*Lupinus angustifolius*). Aust. J. Agric. Res..

[94] Bwye A.M., Proudlove W., Berlandier F.A., Jones R.A.C. (1997). Effects of applying insecticides to control aphid vectors and cucumber mosaic virus in narrow-leafed lupins (*Lupinus angustifolius*). Aust. J. Exper. Agric..

[95] Bwye A.M., Jones R.A.C., Proudlove W. (1999). Effects of different cultural practices on spread of cucumber mosaic virus in narrow-leafed lupins (*Lupinus angustifolius*). Aust. J. Agric. Res..

[96] Thackray D.J., Jones R.A.C., Bwye A.M., Coutts B.A. (2000). Further studies on the effects of insecticides on aphid vector numbers and spread of cucumber mosaic virus in narrow-leafed lupins (*Lupinus angustifolius*). Crop Prot..

[97] Jones R.A.C., Coutts B.A., Latham L.J., McKirdy S.J. (2008). Cucumber mosaic virus infection of chickpea stands: Temporal and spatial patterns of spread and yield-limiting potential. Plant Pathol..

[98] Jones R.A.C. (1993). Effects of cereal borders, admixture with cereals and plant density on the spread of bean yellow mosaic potyvirus into narrow-leafed lupins (*Lupinus angustifolius*). Ann. Appl. Biol..

[99] Jones R.A.C. (1994). Virus Diseases of Lupins.

[100] Jones R.A.C., Coutts B.A., Cheng Y. (2003). Yield limiting potential of necrotic and non-necrotic strains of bean yellow mosaic virus in narrow-leafed lupin (*Lupinus angustifolius*). Aust. J. Agric. Res..

[101] Coutts B.A., Prince R.T., Jones R.A.C. (2009). Quantifying effects of seed-borne virus inoculum on virus spread, yield losses and seed infection in the pea seed-borne mosaic virus—Field pea pathosystem. Phytopathology.

[102] Palukaitis P., Garcia-Arenal F. (2003). Cucumber Mosaic Virus. Descriptions of Plant Viruses No. 400.

[103] Crowley N.C. (1954). Some variables affecting the use of cowpea as an assay host for cucumber mosaic virus. Aust. J. BioI. Sci..

[104] Harvey H.L. (1954). Plant disease—Cucumber mosaic virus. West. Aust. J. Agric. Third Ser..

[105] Latham L.J., Jones R.A.C. (2001). Incidence of virus infection in experimental plots, commercial crops and seed stocks of cool season crop legumes. Aust. J. Agric. Res..

[106] Latham L.J., Jones R.A.C., McKirdy S.J. (2001). Cucumber mosaic cucumovirus infection of cool-season crop, annual pasture, and forage legumes: Susceptibility, sensitivity and seed transmission. Aust. J. Agric. Res..

[107] Aftab M., Freeman A., Davidson J. Pulse virus surveys in south eastern Australia (2003–2004). Proceedings of the Australasian Plant Pathology Society 15th Biennial Conference.

[108] Aftab M., Nancarrow N., Freeman A., Davidson J., Rodoni B., Trębicki P. (2018). Natural infection of cucumber mosaic virus, pea seed-borne mosaic virus and turnip yellows virus in a fenugreek crop (*Trigonella foenum-graecum*). Australas. Plant Dis. Notes.

[109] Association of Applied Biologists (2023). Descriptions of Plant Viruses.

[110] Centre for Agriculture and Biosciences International (2022). Data Sheets. Invasive Species Compendium.

[111] Gladstones J.S. (1967). Uniwhite—A new lupin variety. West. Aust. J. Agric. Fourth Ser..

[112] Jones R.A.C. (1988). Seed-borne cucumber mosaic virus infection of narrow-leafed lupin (*Lupinus angustifolius*) in Western Australia. Ann. Appl. Biol..

[113] McLean G.D., Price L.K. (1984). Virus, Viroid, Mycoplasma and Rickettsial Diseases of Plants in Western Australia.

[114] Bowyer J.W., Keirnan E. (1981). Cucumber mosaic virus in lupin. Australas. Plant Pathol..

[115] Alberts E., Hannay J., Randles J.W. (1985). An epidemic of cucumber mosaic virus in South Australian lupins. Aust. J. Agric. Res..

[116] Jones R.A.C. (1987). Cucumber mosaic virus in lupins. West Aust. J. Agric. Fourth Ser..

[117] Jones R.A.C., Coutts B.A., Kehoe M.A. (2010). Cucumber Mosaic Virus in Lupins.

[118] Wells H.D., Corbett M.K., Forbes I. (1964). Cucumber mosaic virus is seed-borne in blue lupines. Phytopathology.

[119] Lupuwana P., Rybicki E.P., von Wechmar M.B. Cucumber mosaic virus in lupins. Proceedings of the 23rd Congress South African Society for Plant Pathology.

[120] Jones R.A.C., Sward R.J. Seed-borne virus diseases in the Western Australian lupin, subterranean clover and annual medic seed collections and breeding/evaluation programmes. Proceedings of the Workshop on Production, Maintenance and Exchange of Healthy Legume Germplasm.

[121] Geering A.D.W., Randles J.W. Interactions between a seed-borne strain of cucumber mosaic cucumovirus and its lupin host. Ann. Appl. Biol..

[122] Wahyuni W.S., Francki R.I.B. (1992). Responses of some grain and pasture legumes to 16 strains of cucumber mosaic virus (CMV). Aust. J. Agric. Res..

[123] Wahyuni W.S., Dietzgen R.G., Hanada K., Francki R.I.B. (1992). Serological and biological variation between and within subgroup I and II strains of cucumber mosaic virus. Plant Pathol..

[124] McKirdy S.J., Jones R.A.C. (1994). Infection of alternative hosts associated with narrow-leafed lupin (*Lupinus angustifolius*) and subterranean clover (*Trifolium subterraneum*) by cucumber mosaic virus and its persistence between growing seasons. Aust. J. Agric. Res..

[125] Jones R.A.C., McKirdy S.J. (1990). Seed-borne cucumber mosaic virus infection of subterranean clover in Western Australia. Ann. Appl. Biol..

[126] Jones R.A.C. (1991). Losses in productivity of subterranean clover swards caused by sowing cucumber mosaic virus-infected seed. Ann. Appl. Biol..

[127] Pathipanawat W., Jones R.A.C., Sivasithamparam K. (1995). Studies on seed and pollen transmission of alfalfa mosaic, cucumber mosaic and bean yellow mosaic viruses in cultivars and accessions of annual Medicago species. Aust. J. Agric. Res..

[128] Cowling W.A., Gladstones J.S. (2000). Lupin Breeding in Australia. Linking Research and Marketing Opportunities for Pulses in the 21st Century, Proceedings of the Third International Food Legumes Research Conference.

[129] Jones R.A.C. (1991). Reflective mulch decreases the spread of two non-persistently aphid transmitted viruses to narrow-leafed lupin (*Lupinus angustifolius*). Ann. Appl. Biol..

[130] Berlandier F.A., Thackray D.J., Jones R.A.C., Latham L.J., Cartwright L. (1997). Determining the relative roles of different aphid species as vectors of cucumber mosaic and bean yellow mosaic viruses in lupins. Ann. Appl. Biol..

[131] Bwye A.M., Jones R.A.C., Proudlove W. (1995). Management of cucumber mosaic virus in lupins. West. Aust. J. Agric. Fourth Ser..

[132] Wylie S., Wilson C.R., Jones R.A.C., Jones M.G.K. (1993). A polymerase chain reaction assay for cucumber mosaic virus in lupin seeds. Aust. J. Agric. Res..

[133] O’Keefe D.C., Berryman D.I., Coutts B.A., Jones R.A.C. (2006). Absence of seed coat contamination with cucumber mosaic virus allows large-scale detection of seed transmission in lupin seed samples. Proceedings of the 7th Australasian Plant Virology Workshop, Rottnest Island.

[134] O’Keefe D.C., Berryman D.I., Coutts B.A., Jones R.A.C. (2007). Lack of seed coat contamination with cucumber mosaic virus in lupin permits reliable, large-scale detection of seed transmission in seed samples. Plant Dis..

[135] Jones R.A.C. (2005). Patterns of spread of two non-persistently aphid-borne viruses in lupin stands under four different infection scenarios. Ann. Appl. Biol..

[136] Jones R.A.C., Cowling W.A., Neves Martins J.M., Reiraio da Costa M.L. (1994). Screening for resistance to seed transmission of cucumber mosaic virus in *Lupinus angustifolius*. Advances in Lupin Research.

[137] Jones R.A.C., Cowling W.A. (1995). Resistance to seed transmission of cucumber mosaic virus in narrow-leafed lupins (*Lupinus angustifolius*). Aust. J. Agric. Res..

[138] Jones R.A.C., Coutts B.A., Reeve N., Cowling W.A., Buirchell B.J. (1999). Screening for Resistance to Cucumber Mosaic Virus in Lupins. Crop Update Conference, Lupin 1999.

[139] Jones R.A.C., Latham L.J. (1994). Natural resistance to cucumber mosaic virus in broad-leafed lupins. Proceedings of the First Australian Lupin Technical Symposium.

[140] Jones R.A.C., Latham L.J. (1996). Natural resistance to cucumber mosaic virus in lupin species. Ann. Appl. Biol..

[141] Garcıa-Arenal F., Palukaitis P., Mahy B.J., van Regenmortel M.H.V. (2008). Cucumber Mosaic Virus. Desk Encyclopedia of Plant and Fungal Virology.

[142] Jones R.A.C., Buirchell G.M. (2004). Resistance to cucumber mosaic virus in *Lupinus mutabilis* (pearl lupin). Australas. Plant Pathol..

[143] Singh Z., Jones M.G.K., Jones R.A.C. (1998). Effectiveness of coat protein and defective replicase gene-mediated resistance against Australian isolates of cucumber mosaic virus. Aust. J. Exp. Agric..

[144] Liu L., Yang R., Wylie S.J., Li H., Jones M.G.K. (1999). Protection conferred by the cucumber mosaic virus replicase gene in transgenic narrow-leafed lupin. Proceedings of the 15th Meeting International Working Group on Legume Viruses.

[145] Yang R., Liu L., Dwyer G.I., Wylie S.J., Jones R.A.C., Jones M.G.K. (1999). Engineering cucumber mosaic virus resistance in narrow-leafed lupins (*Lupinus angustifolius*) based on a defective replicase gene. Proceedings of the 15th Meeting International Working Group on Legume Viruses.

[146] Jones M.G.K., Wylie S.J., Berryman D., Selladurai S., Brien S.J., Li D., Yang R., Ryan K., Li H., Li L., van Santen E., Wink M., Weissmann S., Romer P. (2000). Biotechnological Tools in Lupin Breeding and Disease Control. Lupin, an Ancient Crop for the New Millennium, Proceedings of the 9th International Lupin Conference, Klink/Muritz, Germany, 20–24 June 1999.

[147] Yang R. (2000). Towards Genetic Engineering Cucumber Mosaic Virus (CMV) Resistance in Lupins. Ph.D. Thesis.

[148] Perry J.N., Bell E.D., Smith R.H., Woiwod I.P. (1996). SADIE: Software to measure and model spatial pattern. Asp. Appl. Biol..

[149] Perry J.N., Winder L., Holland J.M., Alston R.D. (1999). Red-blue plots for detecting clusters in count data. Ecol. Lett..

[150] Thackray D.J., Jones R.A.C. (1999). Forecasting Aphid and Virus Risk in Lupins. Crop Update Conference, Lupin 1999.

[151] Thackray D.J., Hawkes J., Jones R.A.C., O’Neill B. (2000). Forecasting Aphid and Virus Risk in Lupins. Crop Update Conference, Lupin 2000.

[152] Thackray D.J., Diggle A.J., Berlandier F.A., Jones R.A.C., Zalucki M.P., Drew R.A.I., White G.C. (1998). Development of a Simulation Model Forecasting Aphid Outbreaks and Spread of Cucumber Mosaic Virus in Lupins. Pest Management—Future Challenges, Proceedings of the 6th Australasian Applied Entomological Research Conference, Brisbane, Australia, 29 September–2 October 1998.

[153] Thackray D.J., Diggle A.J., Berlandier F.A., Jones R.A.C. (2004). Forecasting aphid outbreaks and epidemics of cucumber mosaic virus in lupin crops in a Mediterranean-type environment. Virus Res..

[154] Thackray D.J., O’Neill B. (2000). Implications of the Green Bridge for Viral and Fungal Disease Carry-Over between Seasons. Crop Update Conference, Lupin 2000.

[155] Hawkes J.R., Jones R.A.C. (2005). Incidence and distribution of barley yellow dwarf virus and cereal yellow dwarf virus in oversummering grasses in a Mediterranean-type environment. Aust. J. Agric. Res..

[156] Coutts B.A., Hawkes J.R., Jones R.A.C. (2006). Occurrence of Beet western yellows virus and its aphid vectors in over-summering broad-leafed weeds and volunteer crop plants in the grainbelt region of south-western Australia. Aust. J. Agric. Res..

[157] Maling T., Diggle A.J., Thackray D.J., Siddique K.H.M., Jones R.A.C. (2008). An epidemiological model for externally sourced vector-borne viruses applied to bean yellow mosaic virus in lupin crops in a Mediterranean type environment. Phytopathology.

[158] (2023). Department of Primary Industries and Regional Development. Western Australian Lupin Industry, Department of Primary Industries and Regional Development, South Perth, Western Australia. https://www.agric.wa.gov.au/investment/western-australian-lupin-industry.

[159] Fletcher A., Lawes R., Weeks C. (2016). Crop area increases drive earlier and dry sowing in Western Australia: Implications for farming systems. Crop Pasture Sci..

[160] Australian Bureau of Agricultural Resource Economics and Sciences (2022). Australian Crop Report, Western Australia.

[161] Jones R.A.C., Coutts B.A. (1996). Alfalfa mosaic and cucumber mosaic virus infection in chickpea and lentil: Incidence and seed transmission. Ann. Appl. Biol..

[162] Freeman A.J., Spackman M.E., Aftab M., McQueen V., King S., van Leur J.A., Loh M.H., Rodoni B. (2013). Comparison of tissue blot immunoassay and reverse transcription polymerase chain reaction assay for virus-testing pulse crops from a South-Eastern Australia survey. Australas. Plant Pathol..

[163] van Leur J.A.G., Aftab M., Manning W., Bowring A., Riley M.J. (2013). A severe outbreak of chickpea viruses in northern New South Wales, Australia, during 2012. Australas. Plant Dis. Notes.

[164] Sharman M., Moore M., Van Leur J., Aftab M., Verrell A. (2014). Viral Diseases of Chickpea—Impact and Management.

[165] Aftab M., Freeman A., Davidson J. Virus diseases in South Australian pulse crops (2005–2006). Proceedings of the 16th Biennial Australasian Plant Pathology Society Conference.

[166] Freeman A.J., Aftab M. Surveying for and mapping of viruses in pulse crops in south-eastern Australia. Proceedings of the 13th Biennial Conference of the Australasian Plant Pathology Society.

[167] Freeman A.J., Aftab M., Dobson V. Surveying for viruses in pulse crops in Victoria. Proceedings of the 8th International Congress of Plant Pathology.

[168] Freeman A.J., Aftab M., McQueen V., Davidson J. The occurrence of common viruses in pulse crops in south eastern Australia. Proceedings of the 15th Biennial Australasian Plant Pathology Society Conference.

[169] Maina S., Zheng L., Rodoni B.C. (2021). Targeted Genome Sequencing (TG-Seq) Approaches to Detect Plant Viruses. Viruses.

[170] Latham L.J., Jones R.A.C., Coutts B.A. (2004). Yield losses caused by virus infection in four combinations of non-persistently aphid-transmitted virus and cool-season crop legume. Aust. J. Exp. Agric..

[171] Freeman A., Aftab M. (2011). Effective management of viruses in pulse crops in south eastern Australia should include management of weeds. Australas. Plant Pathol..

[172] Jones R.A.C. (1988). Seed-borne virus diseases in annual pasture legumes. J. Agric. West. Aust. Fourth Ser..

[173] Swenson K.G., Venables D.G. (1961). Detection of two legume viruses in Australia. Aust. J. Exp. Agric..

[174] Garran J., Gibbs A.J. (1982). Studies on alfalfa mosaic virus and alfalfa aphids. Aust. J. Agric. Res..

[175] Jaspars E.M.J., Bos L. (1980). Alfalfa Mosaic Virus. Descriptions of Plant Viruses No. 229.

[176] Jones R.A.C., Pathipanawat W. (1989). Seed-borne alfalfa mosaic virus infecting annual medics (*Medicago* spp.) in Western Australia. Ann. Appl. Biol..

[177] Jones R.A.C. (1996). Report on Survey for Virus Diseases in crops in the Ord River Irrigation Area, 12–14 August 1996. Northern Australian Quarantine Strategy (NAQS).

[178] van Leur J.A.G., Makkouk K.M., Freeman A., Schilg M.A. Occurrence of viruses in faba bean on the Liverpool Plains, northern New South Wales. Proceedings of the 8th International Congress of Plant Pathology.

[179] Maina S., Zheng L., Kinoti W., Aftab M., Nancarrow N., Trębicki P., King S., Constable F., Rodoni B. (2019). Metagenomic analysis reveals a nearly complete genome sequence of alfalfa mosaic virus from a field pea in Australia. Microbiol. Res. Announc..

[180] Latham L.J., Jones R.A.C. (2001). Alfalfa mosaic and pea seed-borne mosaic viruses in cool season crop, annual pasture, and forage legumes: Susceptibility, sensitivity, and seed transmission. Crop Past. Sci..

[181] McKirdy S.J., Jones R.A.C. (1994). Infection of alternative hosts associated with annual medics (*Medicago* spp.) by alfalfa mosaic virus and its persistence between growing seasons. Aust. J. Agric. Res..

[182] Jones R.A.C. (2004). Occurrence of virus infection in seed stocks and 3-year-old pastures of lucerne (*Medicago sativa*). Aust. J. Agric. Res..

[183] Nutter F.W., Jones R.A.C., Geering A.D.W., Randles J.W., Graetz D., Alberts E.V. (1996). Quantification of alfalfa mosaic virus and cucumber mosaic virus epidemics in the lupin-medic pathosystem. Phytopathology.

[184] Jones R.A.C., Pearce A.R., Prince R.T., Coutts B.A. (2008). Natural resistance to alfalfa mosaic virus in different lupin species. Australas. Plant Pathol..

[185] Stubbs L.L. (1947). A destructive vascular wilt virus disease of broad bean (*Vicia faba* L.) in Victoria. J. Dep. Agric. Vic..

[186] Taylor R.H., Stubbs L.L. (1972). Broad Bean Wilt Virus. Descriptions of Plant Viruses.

[187] Zhou X. (2002). Broad Bean Wilt Virus 2. Descriptions of Plant Viruses.

[188] Makkouk K.M., Kumari S.G., Bos L. (1990). Broad bean wilt virus: Host range, purification, serology, transmission characteristics, and occurrence in faba bean in West Asia and North Africa. Neth. J. Plant Pathol..

[189] Moghal S.M., Francki R.I.B. (1974). Occurrence and properties of broad bean stain virus in South Australia. Aust. J. Biol. Sci..

[190] Randles J.W., Dube A.J. (1977). Three seedborne pathogens isolated from Vicia faba seed imported from the United Kingdom. Australas. Plant Pathol. Soc. Newsl..

[191] Gibbs A.J., Smith H.G. (1970). Broad Bean True Mosaic Virus. Descriptions of Plant Viruses.

[192] Gibbs A.J., Smith H.G. (1970). Broad Bean Stain Virus. Descriptions of Plant Viruses.

[193] Behncken G.M. (1970). The occurrence of peanut mottle virus in Queensland. Aust. J. Agric. Res..

[194] Behncken G.M., McCarthy G.J. (1973). Peanut mottle virus in peanuts, navy beans and soybeans. Qld. Agric. J..

[195] Zanardo L.G., Carvalho C.M. (2017). Cowpea mild mottle virus (*Carlavirus*, *Betaflexiviridae*): A review. Tropical Plant Pathol..

